# Hierarchy Aware CT-Driven Constrained Constructive Optimization: A Framework for Coronary Network Generation

**DOI:** 10.1007/s10439-026-04383-4

**Published:** 2026-09-30

**Authors:** S. L. Vajire, Jenny S. Choy, Ghassan S. Kassab, Lik-Chuan Lee

**Affiliations:** 1 Department of Mechanical Engineering, Michigan State University, East Lansing, MI, USA; 2 California Medical Innovations Institute, San Diego, CA, USA

**Keywords:** Coronary arterial tree generation, Constrained constructive optimization, Diameter-defined Strahler ordering, Coronary morphometry, Subject-specific modeling, CT-based coronary segmentation

## Abstract

**Purpose:**

Subject-specific three-dimensional models of the coronary arterial tree are widely used for simulations of coronary flow and myocardial perfusion. Current image-based reconstructions, however, cannot capture the full microvascular hierarchy, whereas existing synthetic tree generation methods either operate in idealized left ventricular geometries or lack consistency with experimental vessel hierarchy when constrained by anatomy.

**Methods:**

To address these issues, we present a hierarchy-aware CT-driven constrained constructive optimization (HCT-CCO) framework that generates coronary arterial trees as extensions from segmented subject-specific base epicardial coronary networks. The method combines a distance field that restricts candidate segments to the segmented myocardial wall, applies Kamiya-type optimization seeded with HK-type daughter radii and a flow-weighted bifurcation point, and a hierarchy-aware, stage-wise branching schedule that progressively shifts growth toward distal segments as the domain fills. The resulting networks are assigned diameter-defined Strahler orders and analyzed in terms of diameter–order scaling, order–order connectivity, and transmural segment distributions.

**Results:**

We show that the HCT-CCO algorithm is more stable across stochastic realizations and produces connectivity matrices and diameter–order curves that show improved agreement with experimental benchmarks compared to the conventional CCO algorithm. Application to one synthetic case and five subject-specific CT datasets yielded coronary trees spanning diameter-defined Strahler orders 1–11 with monotonic diameter–order trends and mean diameters consistent with measured porcine coronary morphometry and transmural segment distributions across cases.

**Conclusion:**

The HCT-CCO algorithm thus provides a hierarchy-aware, image-constrained approach for generating subject-specific coronary arterial networks that jointly capture experimentally supported diameter–order relations, hierarchical connectivity, and transmural vessel distributions, and offers anatomically grounded substrates for coronary hemodynamic and perfusion modeling.

## Introduction

Faithful anatomical reconstructions of the coronary vasculature are critical for developing fundamental understanding of its physiology, performing detailed mechanistic studies of myocardial perfusion, and developing coronary heart disease treatments [1–3]. Computational models are increasingly used to study multiscale myocardial blood flow regulation and cardiac coronary coupling, including ischemia-related mechanisms and therapy response in settings where microcirculatory measurements are limited *in vivo* [4–7]. Over the past two decades, advances in cardiac imaging and computational modeling have also significantly improved our ability to study the coronary network and circulation *in vivo* [8–10]. Despite the progress achieved with modern imaging techniques, however, current clinical scanners are unable to capture the full extent of intramyocardial microvasculature in the heart wall [11, 12]. As a result, synthetic or generative methods are often used to reconstruct extended or complete coronary trees spanning across multiple spatial scales. Although these methods aim to reproduce physiological branching patterns and realistic diameter scaling, achieving these properties in a subject-specific setting remains challenging.

Early morphometric analyses of the coronary vasculature have shown that the mean vessel diameter increases approximately exponentially with hierarchical order, yielding an approximately linear trend on semi-logarithmic plots. The porcine dataset by [13] remains the widely accepted benchmark for validating such an order-wise scaling relationship and for characterizing coronary branching patterns at the level of connectivity between orders. To facilitate direct comparison across studies, the diameter-defined Strahler (DDS) ordering scheme has been proposed to reduce overlap between adjacent orders [14]. A broad family of constructive growth approaches, including constrained constructive optimization (CCO) variants, has been progressively developed to incorporate additional realism through improved domain representations and organ-specific constraints. Most conventional CCO-based coronary tree generation algorithms, however, have been developed and evaluated in idealized or generic perfusion domains rather than being integrated and driven by subject-specific computed tomography (CT) anatomy [15–17]. While a small number of more recent coronary-focused tree generation algorithms have begun to incorporate image-derived epicardial coronaries, they are typically limited in scope. Specifically, these algorithms are often restricted to a subset of the hierarchy, and do not provide a robust end-to-end continuation in which growth of the distal tree resumes directly from a segmented subject-specific epicardial base tree while preserving proximal anatomy and vessels’ diameter throughout the extension process [18, 19]. In addition, hierarchy-level validation of these algorithms is not consistently reported in a standardized manner, particularly for diameter versus DDS order across the full vessel order range (orders 1–11). Order-to-order connectivity matrices, vessel distribution across the transmural heart wall and stochastic growth repeatability across multiple realizations are also not routinely assessed, even though they are important for establishing whether the generated trees are stable and physiologically consistent under stochastic branching [15–19].

To address these issues, we developed a hierarchy-aware CT-driven constrained constructive optimization (HCT-CCO) framework that generates coronary arterial tree networks as anatomically grounded extensions of subject-specific base coronary macrovascular trees within the CT reconstructed LV geometries. The framework uses a base epicardial coronary tree that is segmented from CT images as the proximal scaffold and introduces hierarchy-aware growth control to stabilize the emerging diameter-order organization under stochastic branching. Bifurcation geometry and vessel diameters are determined through a physiologically motivated local optimization strategy based on flow-dependent constraints and HK-type coronary branching scaling [20], improving the consistency of generated branching structure [14]. Using standardized DDS ordering [14] and experimental morphometry benchmarks [13], we demonstrate that HCT-CCO can reproduce physiological diameter–order relationships and experimentally derived order-to-order connectivity patterns across different reconstructed left ventricular geometries and base epicardial coronary trees. Throughout this work, we refer to the standard CCO formulation introduced by Schreiner et al. [1] and implemented in three dimensions by Karch et al. [15] as conventional CCO and adopt it as the baseline for comparison against the proposed hierarchy-aware framework (HCT-CCO, defined in Sect. “HCT-CCO Algorithm”).

## Methodology

### Pre-processing: From Imaging to Inputs of the HCT-CCO Algorithm

Six datasets were used in this study, with one synthetically generated and the other five derived from CT images of porcine hearts. For datasets derived from the CT images [21], the left ventricular (LV) myocardial wall and large epicardial coronary vessels were manually segmented using 3D Slicer [22] (Fig. 1). These segmentations provide (i) the LV myocardial growth domain and (ii) the initial epicardial base tree geometry used to initialize the HCT-CCO algorithm. Figure 2 shows the overall preprocessing workflow using the synthetic dataset as an illustration. The same processing steps were applied to all the datasets. Specifically, the LV wall segmentation (Fig. 2a) was rasterized and refined to define the final growth domain Ω (Fig. 2c), while the coronary segmentation was cleaned and processed to extract a simplified centerline graph with preserved bifurcations that serve as the epicardial base tree for subsequent growth (Fig. 2f).

#### LV Myocardial Wall Domain

Epicardial and endocardial surfaces were rasterized to generate a volumetric representation of the LV myocardial wall (Fig. 2b). The voxel resolution was selected to capture the overall ventricular geometry and to ensure consistency across datasets. Additional preprocessing steps were applied to produce a watertight and contiguous volume suitable for computing a Euclidean distance transform (EDT) [23]. The segmented surfaces were subsequently converted into a volumetric mask delineating the myocardial wall, which defines the admissible growth region of the coronary vasculature Ω (Fig. 2c). The voxels corresponding to the LV cavity or external space were defined to be inadmissible for growth. A distance transform was evaluated throughout Ω to quantify the local clearance from the epicardial and endocardial boundaries. This distance field supports both model initialization and constraint enforcement during HCT-CCO-based vascular growth by ensuring physiologically realistic spatial confinement.

#### Coronary Vessel Pre-processing and Base-Tree Extraction

Local lumen diameter information derived from the segmented lumen volume was used to obtain initial radius estimates for each vessel segment in the base epicardial coronary network segmented from the CT images [24], which includes the LAD, LCx, and major side branches [13, 25] (Fig. 1). Because segmentation-derived diameter estimates can be locally inconsistent, these radii were subsequently adjusted slightly to ensure global physiological consistency. Small spurious branches arising from segmentation noise or discretization were removed to avoid non-physiological structures that could bias subsequent modeling.

The cleaned vessel was thinned to obtain an accurate centerline representation (Fig. 3b) [26] as applying centerline extraction directly to the full-lumen segmentation often leads to clustered or unstable centerlines [27]. The resulting centerline network was conservatively simplified so that each vessel segment is represented by a minimal number of nodes while preserving vessel trajectories and bifurcation locations (Fig. 3c–d). The final simplified centerline graph, together with the adjusted radii, was used as a base tree for the HCT-CCO algorithm [28].

### HCT-CCO Algorithm

The HCT-CCO algorithm generates a vascular tree using steady, laminar pipe-flow relations and pressure-constrained radius scaling as internal constraints during growth [1]. These relations do not aim to simulate full hemodynamics; rather, they provide a consistency framework that enforces resistance balance and physiological radius scaling compatible with the prescribed perfusion pressure drop as each new terminal branch is accepted.

#### Nomenclature/Symbols

**Table:** 

Symbol	Description
Ω	Admissible LV myocardial domain
EDT	Euclidean distance transform of Ω
$L_{e}$	Segment length
$r_{e}$	Segment radius
$Q_{e}$	Segment flow
$R_{e}$	Segment resistance $\frac{8\mu L}{\pi r^{4}}$
μ	Blood viscosity (constant)
$\Delta p_{e}$	Pressure drop across a segment
$p_{\text{Perf}}$	Perfusion (inlet) pressure
$p_{\text{Term}}$	Terminal pressure
$\Delta p_{\text{root}}$	Perfusion drop $p_{\text{Perf}}-p_{\text{Term}}$
$r_{\text{root}}$	Inlet (root) radius
$\tilde{R}$	Baseline resistance $\frac{8\;\mu L}{\pi }$
${\tilde{R}}_{L},{\tilde{R}}_{R}$	Baseline resistances of daughters
${\tilde{R}}_{\text{down}}$	Parallel baseline of daughters
${\tilde{R}}_{\text{parent}}$	Parent baseline resistance
${\tilde{R}}_{eq}$	Baseline equivalent resistance at root
$R_{eq}$	Physical equivalent $\frac{{\tilde{R}}_{eq}}{r_{\text{root}}^{4}}$
$x_{u},y_{1},y_{2}$	Upstream and daughter nodes
$b,b^{\left(0\right)},b^{*}$	Bifurcation point (current/init/opt)
$l_{0},l_{1},l_{2}$	Parent/daughter segment lengths
$f_{0},f_{1},f_{2}$	Flows in parent and daughters
$r_{0},r_{1},r_{2}$	Parent and daughter radii
ρ	Flow–resistance ratio ${\left(f_{1}{\tilde{R}}_{1}/\left(f_{2}{\tilde{R}}_{2}\right)\right)}^{\frac{1}{4}}$
γ	HK exponent (γ=2.33)
$\beta ,\beta_{1},\beta_{2}$	Radius ratios (child ÷ parent)
$x_{1},x_{2}$	Squared daughter radii $\left(r_{1}^{2},r_{2}^{2}\right)$
$\Delta P_{1},\Delta P_{2}$	Pressure drop proxies along daughters
M	HK-type compliance factor
$R_{1},R_{2}$	Residuals (Kamiya stationarity)
Π	Growth fraction $\frac{k}{N_{\text{target}}}$
s(Π)	Restriction level ∈{0,0.5,1}
$k_{\text{term}}$	Number of terminals in a subtree
$q_{\text{term}}$	Flow per terminal
$Q_{\text{total}}$	Total perfusion flow
$N,N_{\text{target}}$	Current / target number of terminals
n	DDS order index (Eq. 12)
${\overset{‾}{D}}_{n},\sigma_{n}$	Mean, std of diameters in order n
$D_{1(n)}^{\prime },D_{2(n)}^{\prime }$	DDS transition thresholds
λ	Diameter scaling ratio
k	DDS order index (affine step)
$d,d^{\prime }$	Segment / transformed diameter
$\mu_{k}^{\text{sim}},\sigma_{k}^{\text{sim}}$	Simulated mean, std in order k
$\mu_{k}^{\text{ref}},\sigma_{k}^{\text{ref}}$	Measured mean, std in order k

#### Boundary-Aware Domain Integration for HCT-CCO Growth

The LV mask and its associated distance field were integrated into the HCT-CCO solver to regulate terminal placement and ensure that vascular growth remains confined to the myocardial wall. Terminal candidates were sampled within the admissible region Ω and retained only if they satisfy a prescribed clearance $d_{\text{min}}$ from the epicardial and endocardial surfaces. Evaluating these distances on the continuous field, rather than on discrete voxel boundaries, provides subvoxel geometric fidelity and reduces boundary artifacts commonly encountered in conventional CCO algorithm implementations.

To assess the feasibility of a proposed segment, the straight-line path between its endpoints was discretized, and the wall distance was evaluated at each location using trilinear interpolation of the EDT [23]. Segments were accepted only when all sampled points met the clearance requirement; otherwise, the proposed segment was rejected, and a new candidate segment was generated. By embedding these boundary-aware constraints throughout sampling, bifurcation placement, and segment validation, the method provides an anatomically grounded extension of the conventional CCO algorithm, enabling vascular growth that conforms naturally to the LV geometry while preserving the incremental structure of the algorithm.

#### Hydraulic Flow Relations

Each vessel segment was modeled as a cylindrical conduit characterized by its length $L_{e}$, radius $r_{e}$, volumetric flow rate $Q_{e}$, and hydraulic resistance $R_{e}$. Segment lengths were computed from the Euclidean distances between connected nodes, and the radii were updated immediately after each accepted growth step to keep the geometric and hydraulic properties synchronized throughout the growth of the tree. Under the assumption of steady laminar flow, Poiseuille’s law defines the pressure–flow relationship (Eq. 1) [29]:

(1) $$R_{e}=\frac{8\mu L_{e}}{\pi r_{e}^{4}},\Delta p_{e}=R_{e}Q_{e}.$$

with viscosity μ treated as constant.

Following the addition of a new terminal segment, hydraulic resistances were updated incrementally along the path from the new terminal to the root, with only the affected segment and its upstream ancestors requiring recomputation of $L_{e}$ and $R_{e}$ (Eq. 1). All other branches retained their previously valid hydraulic state. This incremental update improves upon the conventional global-recalculation approach by preserving identical fluid physics while avoiding unnecessary recomputation across the rest of the tree. In doing so, the method reduces numerical drift, improves computational efficiency, especially in large networks where the update cost grows primarily with tree depth, and maintains close synchrony between geometry and flow as the vascular tree expands [1, 8].

#### Inlet Radius Adaptation and Downstream Propagation

Following the incremental hydraulic update described above, the inlet and downstream vessel radii were adjusted to maintain consistency between geometry, flow, and the prescribed perfusion pressure drop [1, 29]. At each iteration, the inlet radius is updated by enforcing $\Delta p_{\text{root}}=R_{\text{root}}Q_{\text{root}}$, with $R_{\text{root}}=\frac{{\tilde{R}}_{eq}}{r_{\text{root}}^{4}}$, where ${\tilde{R}}_{eq}$ is the radius-independent baseline equivalent resistance of the root-plus-downstream network. To compute ${\tilde{R}}_{eq}$ efficiently, we separate each segment resistance into a radius-independent baseline and an inverse fourth-power radius scaling, i.e., ${\tilde{R}}_{e}=\frac{8\mu L_{e}}{\pi }$, so that $R_{e}=\frac{{\tilde{R}}_{e}}{r_{e}^{4}}$. The network baseline equivalent resistance ${\tilde{R}}_{eq}$ is then obtained by combining segment baselines through standard series and parallel reductions over the current tree topology. The inlet radius then follows as,

(2) $$r_{\text{root}}=\text{min}\left(1.7mm,{\left(\frac{{\tilde{R}}_{eq}Q_{\text{root}}}{\Delta p_{\text{root}}}\right)}^{\frac{1}{4}}\right),\Delta p_{\text{root}}=p_{\text{Perf}}-p_{\text{Term}}.$$

This formulation reduces to the classical single-segment Poiseuille relation when the equivalent resistance corresponds to the root segment alone. To avoid unrealistically large proximal radius during early stages of growth, the inlet radius was constrained not to exceed 1.7 mm (Eq. 2), corresponding to a lumen diameter of 3.4 mm. This bound is consistent with measured proximal epicardial coronary calibers, in which the proximal left anterior descending and left circumflex arteries exhibit diameters of 3.7 ± 0.4 mm and 3.4–4.2 mm, respectively, in normal human coronaries [25], and with the diameter of the largest diameter-defined Strahler order reported in the porcine morphometric benchmark [13]. This physiologic cap limits excessive dilation near the root while preserving the intended pressure–radius relationship. Following inlet adaptation, downstream radii were updated recursively using bifurcation-derived radius ratios, with a taper-floor factor of 0.7 applied to maintain smooth diameter transitions [30]. This value lies immediately below the symmetric daughter-to-parent diameter ratio predicted by the HK branching law adopted here (γ=2.33; ratio ≈ 0.74) [20] and by Murray’s law (≈ 0.79) [29] and is consistent with the empirical coronary bifurcation relation of Finet et al., in which the parent diameter equals 0.678 times the sum of the two daughter diameters [36]. It therefore prevents the recursive radius update from producing non-physiologic over-tapering while leaving physiologically normal bifurcations unchanged. Together, these updates support stable proximal behavior and ensure a coherent evolution of radius, pressure, and flow throughout tree growth.

#### Local Geometric Optimization of Bifurcations

Each proposed terminal addition undergoes a local geometric optimization that adjusts the daughter radii and bifurcation location to satisfy flow partitioning and minimize energy expenditure [1, 8, 30]. The local bifurcation geometry and optimization procedure are illustrated in Fig. 4.

In conventional CCO implementations, the bifurcation point is typically initialized at the geometric midpoint of the host segment $\left(t_{m}=0.5\right)$ with symmetric daughter radii, providing no flow-informed guidance to the nonlinear solver [1, 8]. As a result, the optimization may begin far from the physically optimal configuration, leading to slow convergence, increased iteration counts, and convergence to poorly conditioned or degenerate states under strong flow asymmetry. By contrast, the present framework introduces analytic, flow-based initialization of both junction location and daughter radii, linking flow, radius, and geometry through an HK-type branching relation and a flow-weighted junction position [20, 29, 30]. This yields a physically consistent starting point that places the solver close to the true optimum, thereby accelerating convergence, improving numerical stability, and avoiding such pathological solutions.

Considering a host segment with upstream endpoint $x_{u}$, and daughter endpoints $y_{1}$ (new terminal) and $y_{2}$ (existing downstream node, denoted $x_{v}=y_{2}$), the initial bifurcation point $b^{\left(0\right)}$ is defined as a flow-weighted barycentric combination of these three nodes:

(3) $$b^{\left(0\right)}=\frac{f_{0}x_{u}+f_{1}y_{1}+f_{2}y_{2}}{2f_{0}},$$

where $f_{0},f_{1},f_{2}$ are the flows through the parent and daughter segments, respectively, satisfying $f_{0}=f_{1}+f_{2}$ by conservation. This positions the initial junction toward the higher flow branch and closer to the true optimum position. Throughout the optimization, b denotes the current bifurcation position, initialized at $b^{\left(0\right)}$ and iteratively refined until convergence to the optimal configuration $b^{*}$. The corresponding segment lengths are:

(4.1) $$l_{0}(b)=\left\|b-x_{u}\right\|,$$

(4.2) $$l_{1}(b)=\left\|b-y_{1}\right\|,$$

(4.3) $$l_{2}(b)=\left\|b-y_{2}\right\|.$$

Given the parent radius $r_{0}$ and daughter flows $f_{1}$ and $f_{2}$, an initial flow ratio

(5) $$\rho ={\left(\frac{f_{1}}{f_{2}}\right)}^{\frac{1}{4}}$$

is used to define trial radius ratios $\beta_{1}$ and $\beta_{2}$, yielding initial daughter radii:

(6.1) $$r_{1}^{\left(0\right)}=r_{0}\beta_{1},r_{2}^{\left(0\right)}=r_{0}\beta_{2},$$

where

(6.2) $$\beta_{1}={\left[\frac{1}{1+\rho^{\gamma }}\right]}^{\frac{1}{\gamma }},\beta_{2}={\left[\frac{1}{1+\rho^{-\gamma }}\right]}^{\frac{1}{\gamma }},$$

and γ is the HK-type bifurcation exponent with a value γ=2.33 [20]. With the initial bifurcation position $b^{\left(0\right)}$ and daughter radii ${{\text{r}}_{1}}^{\left(0\right)},{{\text{r}}_{2}}^{\left(0\right)}$ established, the optimization seeks to minimize the total hydraulic resistance of the local bifurcation. This is achieved by iteratively adjusting the daughter radii and bifurcation position until the pressure drops along both daughter paths are balanced according to the Kamiya–Togawa optimality conditions [30]. For flows $f_{0},f_{1},f_{2}$, lengths $l_{0},l_{1},l_{2}$, and radii $r_{0},r_{1},r_{2}$, local pressure drop proxies along the two daughter paths are defined as:


(7) $$\Delta P_{1}=\frac{f_{0}\;l_{0}}{r_{0}^{4}}+\frac{f_{1}\;l_{1}}{r_{1}^{4}},\Delta P_{2}=\frac{f_{0}\;l_{0}}{r_{0}^{4}}+\frac{f_{2}\;l_{2}}{r_{2}^{4}}.$$

With solver variables $x_{1}=r_{1}^{2}$ and $x_{2}=r_{2}^{2}$ (squared daughter radii), the HK-type compliance factor is defined as:


(8) $$M={\left[f_{0}\left(\frac{x_{1}^{\frac{3+\gamma }{2}}}{f_{1}}+\frac{x_{2}^{\frac{3+\gamma }{2}}}{f_{2}}\right)\right]}^{\frac{2}{3}}.$$

Using these definitions, the residuals minimized by the Ceres solver are derived from the stationarity conditions of the Kamiya hydraulic cost functional:

(9.1) $$R_{1}=\Delta P_{1\;}x_{1}^{2}M-f_{0\;}l_{0}x_{1}^{2}-f_{1\;}l_{1}M,$$

(9.2) $$R_{2}=\Delta P_{2\;}x_{2}^{2}M-f_{0\;}l_{0}x_{2}^{2}-f_{2\;}l_{2}M.$$

Since the segment lengths depend on b via (Eqs. 4.1–4.3), the residuals depend on both the bifurcation position and the daughter radii: $R_{i}=R_{i}\left(b,x_{1},x_{2}\right)$. Small residuals correspond to configurations in which the effective resistances along both daughter paths are jointly consistent with the HK-type branching law and Kamiya optimality conditions.

Starting from the initial estimate $b^{\left(0\right)}$ given by (Eq. 3) and the initial daughter radii from (Eq. 6.1), the solver iteratively refines the bifurcation position b. At each iteration, the segment lengths are recomputed via (Eqs. 4.1–4.3) using the current bifurcation position b, the pressure drop proxies and branching law factor are evaluated from (Eq. 7) and (Eq. 8), and the residuals R_1_ and R_2_ are minimized to obtain updated daughter radii using (Eqs. 9.1–9.2). The bifurcation position b is then updated as a weighted combination of the three endpoints, based on squared-radius-to-length ratios:

(10) $$b=\frac{\frac{x_{u\;}r_{0}^{2}}{l_{0}}+\frac{y_{1}{\;r}_{1}^{2}}{l_{1}}+\frac{y_{2\;}r_{2}^{2}}{l_{2}}}{\frac{r_{0}^{2}}{l_{0}}+\frac{r_{1}^{2}}{l_{1}}+\frac{r_{2}^{2}}{l_{2}}}.$$

This iterative process continues until convergence, yielding the final optimized configuration $b^{*}$. Candidate solutions are accepted only if they satisfy taper-floor limits, spacing constraints, segment length caps, and the myocardial domain admissibility rules. In particular, vessel overlaps are prevented by a radius-aware intersection test: each candidate segment, defined by its centerline and radius, is checked against all existing segments as a tube-to-tube intersection, and the bifurcation is accepted only if none of its segments intersects an existing vessel. This test is applied both when selecting the connection and after the local optimization, and terminal placement additionally enforces a minimum clearance scaled by the local vessel radius. The analytic initialization based on flow-weighted barycentric positioning and radius seeding substantially improves convergence stability, reduces iteration counts, and prevents degenerate radius ratios under strong flow asymmetry, while retaining the conventional CCO objective of minimizing hydraulic resistance along the tree.

#### Tree Initialization, Reconstruction, and Extension

Following preprocessing, each tree reconstruction is initialized from a segmentation-derived coronary base tree together with its voxelized LV domain. The base tree provides the proximal architecture resolved in CT, represented as a rooted, directed graph with spatially registered node coordinates and lumen-derived surrogate radii. Because this graph is already embedded within the admissible myocardial region and structurally formatted to satisfy the geometric and topological requirements of the CCO solver, the algorithm does not regenerate the visible vasculature. Instead, it continues growing from the base tree, extending the network into intramyocardial territories below imaging resolution [18, 31]. This resume-from-segmentation capability extends the conventional CCO algorithm, which is typically initialized from a synthetic root, allowing subject-specific coronary tree extension from imaging-derived anatomy [18]. This strategy enables physiological distal synthesis while preserving the extracted macrovascular geometry, enforces strict spatial confinement within the myocardial wall, and maintains hemodynamic consistency with the evolving flow and radius field across all subsequent growth iterations [31]. Together, these properties establish the foundation for subject-specific coronary tree extension in the subsequent growth stages.

#### Hierarchy-Aware Branching Strategy

In conventional CCO growth, each new terminal segment may, in principle, be connected to any existing segment in the tree, with the final attachment determined by optimization of the governing cost functional [15–17]. The set of admissible branching locations remains unchanged throughout growth, relying on the optimization process alone to determine where new bifurcations arise. In the present framework, this globally admissible branching space is progressively constrained as growth proceeds, so that branch placement is regulated in a stage-dependent manner while retaining the same cost-based selection criterion within each admissible stage.

When growth is initialized from the CT-derived segmentation, the available base tree typically contains proximal and mid-order vessels. In this setting, a hierarchy-aware branching strategy is employed to guide the extension in a physiologically consistent manner. An initial phase of relaxed branching eligibility allows coherent densification of intermediate generations; branching is then progressively restricted toward distal attachment as growth advances (Fig. 5).

Eligibility of branching is governed by a progress-dependent restriction schedule defined in terms of the normalized growth fraction:

(11) $$\Pi =\frac{k}{N_{\text{target}}},$$

where k denotes the current number of terminal vessels and $N_{\text{target}}$ is the prescribed target number of terminals for the simulation. A discrete restriction level s(Π)∈{0, 0.5, 1} specifies the subset of existing segments eligible for new bifurcations. The restriction schedule is defined as:

- s=0 for 0≤Π<0.10: unrestricted branching,
- s=0.5 for 0.10≤Π<0.35: branching limited to terminal segments and a finite band of their ancestors,
- s=10 for 0.35≤Π≤1: branching restricted to terminal segments and their immediate parents.

The stage-wise restriction pattern was motivated by hierarchical branching trends reported in the coronary connectivity matrix [13], which characterizes how vessels at different hierarchical levels distribute their branching relationships. These data indicate broadly distributed connectivity in proximal vessels, a narrower but still multi-level attachment range in mid-order generations, and increasingly localized branching behavior in distal arterioles. The three-stage restriction schedule was designed to reflect this progressive narrowing of permissible branching relationships, thereby inducing a physiologically consistent transition from proximal organization to distal refinement during growth.

The specific transition points follow the vessel-count distribution of the coronary tree. Because vessel number increases geometrically toward smaller orders [13], distal arterioles constitute a large majority of segments, so the schedule reserves only the initial 10% of growth for the proximal scaffold and devotes the remaining growth, roughly two thirds of all terminals from the 35% point onward, to distal-only branching. The thresholds therefore delimit broad growth phases rather than sharp switching points; cost-based optimization continues to select among the admissible connections within each phase, so they act as soft transitions rather than precise constraints.

#### Growth Execution and Reproducibility

At each growth iteration, candidate terminal locations are sampled from the distance field-defined admissible myocardial domain, and candidate attachment segments are selected according to the hierarchy-aware branching restrictions described above. For each admissible candidate, a local bifurcation configuration is evaluated using the geometric optimization framework introduced previously, initialized with HK-type radius and position seeds, and solved using a Kamiya-style formulation [30]. Proposed branches are incorporated into the tree only if all geometric, hydraulic, and domain constraints are satisfied.

Upon acceptance of a new branch, the vascular state is updated to maintain consistency between geometry and hemodynamics. Hydraulic resistances were recomputed incrementally along the affected path from the new terminal to the root, while flows were maintained using the terminal-count-based prescription, in which each terminal receives equal flow $Q_{\text{term}}=\frac{Q_{\text{total}}}{N}$, and any segment carries flow proportional to the number of terminals in its subtree. The equal per-terminal flow is obtained by dividing the prescribed total coronary inlet flow by the number of terminals, and flows are not weighted by the myocardial volume each terminal supplies. Inlet and downstream vessel radii were then adapted to remain compatible with the prescribed perfusion pressure drop under this fixed flow allocation. This sequence is repeated until the target number of terminal vessels is reached (Fig. 6).

Throughout growth, the solver preserves the complete geometric and hemodynamic state of the evolving tree, enabling simulations to be resumed or further extended without reinitialization. All stochastic components are controlled through fixed random seeds, rendering the growth process deterministic for a given initial tree and seed value. These properties support reproducibility analyses, ensemble generation, and controlled refinement of subject-specific vascular networks. The principal parameters of the HCT-CCO algorithm, their selected values, and the rationale for each are summarized in Table 1.

### Postprocessing

#### Diameter-Defined Strahler (DDS) Ordering

To obtain a physiologically meaningful hierarchy of vessel sizes, we applied a DDS ordering, which augments the classical topological Strahler convention by incorporating measured vessel diameters. Purely topology-based ordering can assign identical orders to vessels with substantially different diameters, particularly in asymmetric bifurcations, leading to overlap in order-wise morphometric statistics. The DDS ordering framework [14, 32] addresses this limitation by redefining order boundaries using empirical diameter distributions, thereby coupling hierarchical order more directly to vessel geometry.

Each vessel segment was first assigned an initial Strahler order based on tree topology. For each order n, the mean and standard deviation of vessel diameters were computed as ${\overset{‾}{D}}_{n}$ and $\sigma_{n}$. Adjacent orders were then separated by two transition thresholds,

(12.1) $$D_{1(n)}^{\prime }=\frac{1}{2}\left[\left({\overset{‾}{D}}_{n-1}+\sigma_{n-1}\right)+\left({\overset{‾}{D}}_{n}-\sigma_{n}\right)\right],$$

(12.2) $$D_{2(n)}^{\prime }=\frac{1}{2}\left[\left({\overset{‾}{D}}_{n}+\sigma_{n}\right)+\left({\overset{‾}{D}}_{n+1}-\sigma_{n+1}\right)\right],$$

such that a vessel with a diameter $D_{i}$ was assigned to order n if $D_{1(n)}^{\prime }<D_{i}\leq D_{2(n)}^{\prime }$. Order assignments and thresholds were updated iteratively until fewer than 1% of the vessels changed order between successive iterations, indicating convergence of the diameter hierarchy. This diameter-based refinement substantially reduced overlap between adjacent orders and yielded a monotonic order–diameter relationship consistent with empirical coronary morphometry, typically of the form ${\overset{‾}{D}}_{n}\propto \lambda^{n}$ with λ≈1.6-1.7.

This combined approach, initial Strahler’s ordering followed by diameter-defined refinement, prevents under-ordering of large proximal segments while maintaining coherence between diameter scaling and branching topology [14, 32]. The resulting DDS ordering provided a stable, physiologically grounded classification of the coronary tree with well-separated diameter distributions across orders. This ordering formed the basis for subsequent analyses of diameter scaling, length ratios, and branching statistics in both synthetic and subject-specific coronary networks. Figure 7 illustrates the centerline graph with radius values obtained from the line graph generation pipeline and the corresponding vessel order distribution after applying diameter-defined Strahler ordering.

#### Order-Wise Affine Diameter Transformation

After tree growth and DDS ordering, we apply an order-wise affine diameter transformation as the final step in the generation pipeline to define the final generated coronary tree. In this operation, the branching topology and DDS ordering labels are fixed, and the diameter field is updated via an order-conditioned affine remapping that enforces prescribed order-wise morphometric constraints derived from experimental data. Each vessel segment is characterized by an assigned order k and a simulated diameter d. For a given order k, the empirical mean and standard deviation of the simulated diameters, $\mu_{k}^{\text{sim}}$ and $\sigma_{k}^{\text{sim}}$, are compared with measurements $\mu_{k}^{\text{ref}}$ and $\sigma_{k}^{\text{ref}}$. Diameters are then prescribed by an order-wise affine transform applied to every vessel in that order, defined directly as

(13) $$d^{\prime }=\mu_{k}^{ref}+\frac{\sigma_{k}^{ref}}{\sigma_{k}^{sim}}\left(d-\mu_{k}^{sim}\right),$$

This expression is obtained by requiring that, when applied to all vessels of order k, the transformed diameters reproduce the measured mean $\mu_{k}^{\text{ref}}$ and standard deviation $\sigma_{k}^{\text{ref}}$. Because the scaling factor $\frac{\sigma_{k}^{\text{ref}}}{\sigma_{k}^{\text{sim}}}$ is strictly positive, the mapping is strictly monotone increasing in d and the relative ranking of diameters within a given order is preserved. The transformation is applied at the level of individual vessels: for each segment, the transformed diameter is computed from its initial diameter and DDS order using the corresponding order-wise parameters. The branching pattern and DDS ordering labels remain unchanged throughout, so the procedure acts solely as an order-wise affine transformation of the diameter scale on the existing hierarchy.

#### Statistical Analysis

Comparisons between the conventional CCO and HCT-CCO frameworks were performed across ten independent stochastic realizations generated on the same LV domain. For each realization, the connectivity F1 score was computed against the binarized literature connectivity matrix [13], and the order-wise diameter deviation was quantified as the mean absolute difference between simulated and measured log_10_ diameters between orders 1 and 11. The two frameworks were compared using the two-sided Mann–Whitney U test, and effect sizes were reported as Cliff’s delta, for which a value of 1.0 indicates complete separation of the two distributions. Significance was set at p<0.05.

#### Hemodynamic assessment

The hemodynamic plausibility of the generated networks was assessed with a steady-state, lumped-parameter resistive network model. Each vessel segment was represented by its Poiseuille resistance, $R=8\mu \text{L}/\left(\pi {\text{r}}^{4}\right)$, and each terminal was coupled to a distal microvascular resistance representing the unresolved downstream vasculature. A perfusion pressure of 100 mmHg was prescribed at the inlet, and the total inlet flow was set to Q=q⋅ρ⋅V, where V is the segmented myocardial volume, ρ≈1.05g/mL is the myocardial tissue density, and q=3.5mL/min/g is a physiological vasodilated perfusion density, so that mean perfusion over the segmented mass equals q. The resulting linear network was solved for segment flows and pressures, and wall shear stress was computed as $\tau =4\mu Q/\left(\pi {\text{r}}^{3}\right)$.

## Results

### Comparison Between Conventional CCO and HCT-CCO Frameworks on a Synthetic LV Geometry

Coronary arterial trees generated using the conventional CCO algorithm and the HCT-CCO framework on an identical LV domain (Case 1) were compared. Qualitatively, the conventional CCO trees filled the myocardial domain but did not reproduce a recognizable LAD or LCx architecture, distributing segments to fill the available space rather than following the hierarchical trunk-and-branch organization of the HCT-CCO networks. The comparison is organized into two parts. First, we examine the order–order connectivity matrix of a representative tree from each formulation and compare these patterns with those obtained from the experiment [13]. The connectivity matrix encodes the presence or absence of a branch from a parent order m to a daughter order n, yielding a binary adjacency pattern in the order space. Second, we analyze the diameter–order relationships produced by each formulation across ten independent stochastic realizations and summarize the associated order statistics to assess the robustness of the hierarchical organization under repeated growth on the same LV geometry.

#### Order–Order Connectivity Pattern at Diameter-Defined Strahler’s Ordering Level

Figure 8 shows a comparison of the directed order–order connectivity matrices constructed from trees generated by the conventional CCO algorithm, the HCT-CCO algorithm, and that derived from experimental data [13]. The results show that the connectivity matrix obtained from the conventional CCO algorithm contains a larger set of parent–daughter order pairs than that from the experimental reference, particularly additional connections from low orders to higher daughter orders. The connectivity matrix from the conventional CCO algorithm also omits several order combinations present in the experimental reference. In contrast, the connectivity matrix obtained from the HCT-CCO framework exhibits a banded near-diagonal structure that closely follows the experimental data, with only a small number of deviations. These differences are highlighted in the non-overlap plots, where the conventional CCO algorithm shows multiple false-positive and false-negative edges relative to the reference, whereas the HCT-CCO framework exhibits only a few mismatched order pairs.

To quantify agreement with the experimental connectivity pattern, each possible parent–daughter order pair was treated as a binary edge and classified as true positive, false positive, or false negative with respect to the literature matrix. For the representative trees shown in Figure 8, the conventional CCO algorithm achieves a precision of 0.833, a recall of 0.816, and an F1 score of 0.825, while the HCT-CCO framework yields a precision of 0.978, a recall of 0.918, and an F1 score of 0.947. To confirm that this improvement was consistent rather than specific to a single tree, the connectivity F1 score was computed for each of ten independent realizations per framework. The HCT-CCO framework yielded a significantly higher F1 score than conventional CCO (0.95 ± 0.01 versus 0.85 ± 0.02; two-sided Mann–Whitney U test, p<0.001; Cliff’s delta = 1.0, indicating that every HCT-CCO realization exceeded every conventional CCO realization), with mean precision and recall of 0.98 and 0.93 versus 0.84 and 0.85, respectively (Fig. 10a). These metrics indicate that the branching rules in the HCT-CCO framework generate an order–order connectivity pattern that is closer to the experimental measurements compared to that obtained from the conventional CCO algorithm.

#### Diameter–Order Relationships Under Stochastic Growth

Figure 9 shows a comparison of the diameter–order hierarchies from 10 independent stochastic realizations using the conventional CCO algorithm and the HCT-CCO algorithm on the same LV domain. Hierarchical development is highly variable and does not yield a reproducible diameter–order structure across ten realizations generated with the conventional CCO algorithm. Specifically, the diameter–order curves exhibit irregular progression across successive orders, and the maximum order attained varies appreciably between simulations. In some realizations, the hierarchy extends beyond the range reported in the experimental measurements [13], whereas in others it terminates at lower orders. In contrast, the HCT-CCO framework exhibits markedly more consistent behavior, where the ten realizations form a tightly clustered diameter–order hierarchy that is closely aligned with the experimental measurements [13]. The diameter–order curves obtained from the ten realizations remain closely aligned and monotone across orders, with substantially reduced inter-run dispersion and a stable maximum order. For each order, the mean diameters from different realizations lie close to the experimental values, with only small deviations within the error bars of the reference data, and the order-wise dispersion remains modest even at higher orders. Overlaying the ensemble-averaged diameter–order curves and the variability envelopes of both algorithms together with experimental measurements, shows that the trees generated by the conventional CCO algorithm display broad dispersion and irregular order-wise progression, whereas those generated by the HCT-CCO framework follow a coherent monotonic trend with markedly reduced spread across realizations. This behavior indicates that the HCT-CCO formulation yields a more reproducible diameter–order structure that is closer to the experiments with less stochastic variability than the conventional CCO algorithm. To quantify these trends, the order-wise diameter deviation from the porcine benchmark was computed for each realization as the mean absolute log__1__0__ difference across orders 1–11. This deviation was substantially smaller for HCT-CCO than for conventional CCO (0.034 ± 0.004 versus 0.998 ± 0.071; two-sided Mann–Whitney U test, p<0.001; Cliff’s delta = 1.0), corresponding to agreement within approximately 8 percent for HCT-CCO compared with roughly one order of magnitude for conventional CCO. The across-realization spread of this deviation was approximately eighteen-fold smaller for HCT-CCO (standard deviation 0.004 versus 0.071), providing a quantitative measure of the reduced stochastic variability evident in Fig. 9 (Fig. 10b).

### Subject-Specific LV Coronary Network Generation

Figure 11 shows the coronary trees generated by the HCT-CCO algorithm for all six cases (Cases 1–6, panels a–f). These include one synthetic case (Case 1) and five subject-specific LV geometries (Cases 2–6) [21]. In all cases, the initial base tree, segmented from the CT images, consists of epicardial vessels of the LAD and LCx branches, corresponding to Strahler orders 6–11, from which the intramural network was subsequently developed. Testing each segment against its spatial neighbors for tube-to-tube intersection confirmed that the generated trees are essentially free of vessel overlaps. In all six cases, crossings between topologically unrelated vessels involved fewer than 0.1% of segments and were of small magnitude (median ~80μm, maximum ~0.3 mm).

#### Diameter–Order Relationships in Subject-Specific LV Coronary Trees

Figure 12 shows the relationship between vessel diameter and DDS order for each of the six cases. In all cases, mean vessel diameter increases monotonically with order and the diameter–order curves follow an approximately linear trend on semi-logarithmic axes over orders 1–11, consistent with the measured quasi-exponential geometric scaling of diameter with hierarchical level. Across the six networks, mean diameters span roughly 0.008–3 mm from the smallest to the largest orders, and the narrow separation among the case curves indicates low inter-case variability in the diameter–order relationship despite differences in LV geometry. The resulting diameter–order curves are in close agreement with the experimental data [13], the arterial hierarchy extends from DDS order 1 to 11, matching the documented order range and indicating a consistent attainment of the expected hierarchical depth across all cases.

#### Transmural Vessel Distribution

Table 2 shows the spatial distribution of vessel segments across the LV wall for all six cases. Across these networks, the endocardial layer (inner 50% of wall thickness) contains 47.87% of the total number of leaf vessels, whereas the epicardial layer (outer 50%) contains 52.13%. This pattern is consistent with a slightly higher concentration of vessels toward the outer wall, indicating a mild epicardial predominance in the transmural distribution of terminal branches. The reported quantity is the percentage of terminal (order 1, leaf) vessels located in the inner and outer halves of the wall, each half defined by equal transmural volume, rather than a vessel density or vascular-volume measure

### Hemodynamic Plausibility

Figure 13 shows the hemodynamic response of the generated network as a function of vessel diameter. With total flow calibrated to a physiological perfusion density (3.5 mL/min/g, within the reported vasodilated coronary range), wall shear stress remained approximately uniform across vessel calibers, with a median of 11 dyn/cm^2^ (Fig. 13a), consistent with the near-constant wall shear stress maintained in coronary vessels. Segment flow increased with vessel diameter following the expected power-law relationship (Fig. 13b). Together, these results indicate that the generated network is hemodynamically plausible and a suitable substrate for downstream flow and perfusion modeling.

### Computational Performance

Generating a complete network of roughly 4 × 10^4^ vessel segments (about 2 × 10^4^ terminals) required on the order of 20 hours per case on a single processor, with a peak memory footprint of approximately 50 MB. Because terminals are added sequentially and the vascular state is updated after each addition, runtime increased super-linearly with terminal count, from approximately one hour at 5 × 10^3^ terminals to approximately five and twenty hours at 1 × 10^4^ and 2 × 10^4^ terminals, respectively. Memory usage, by contrast, was dominated by the voxelized myocardial domain and its distance field rather than by the vascular tree and remained essentially constant as the number of terminals increased. All six subject-specific cases and the ten stochastic realizations completed without growth failure, reaching the full diameter-defined Strahler range in every case.

## Discussion

This study presents a novel HCT-CCO algorithm that generates coronary arterial tree networks from subject-specific base coronary epicardial trees (and LV geometries) that are consistent with the hierarchical organization, order–order connectivity, and order-wise diameter relationships measured in experiments [13]. Using a synthetic LV domain, we first showed that the framework yields a reproducible diameter–order hierarchy under stochastic growth, in contrast to a conventional CCO algorithm in which hierarchical depth, diameter–order trajectories, and DDS order assignment vary substantially between each realization. On the same domain, we then demonstrated that the order–order connectivity pattern produced by the HCT-CCO framework shows close agreement with the experimentally measured coronary connectivity matrix, with markedly fewer spurious or missing order pairs than the conventional formulation. Across one synthetic and five subject-specific LV geometries, the HCT-CCO algorithm produces consistent diameter–order scaling across DDS order 1–11 with perorder diameter statistics aligned with experimental coronary morphometry [13].

### Comparison with Other CCO-Based Coronary Tree Generation Algorithms

Conventional CCO formulations were first developed for generic three-dimensional perfusion domains and then extended to idealized organ geometries. In simple convex volumes, Karch et al. [15] generated large arterial trees and showed that model predicted diameter versus level statistics that follow the experimental corrosion cast data of [33–35]. They later used such trees with different terminal flow prescriptions to explore myocardial perfusion heterogeneity, demonstrating that equal terminal flows reproduce physiological ranges of fractal dimension and nearest-neighbor correlation while Voronoi weighted flows produce patterns more consistent with hypoperfusion [15, 16, 33]. Schreiner et al. [17] then introduced a domain potential to confine CCO tree growth to analytically defined left ventricular wall-like shells, allowing non-uniform transmural perfusion densities while preserving classical morphometric trends in an idealized LV geometry. Taken together, these studies established that CCO can generate realistic segment diameter scaling and global perfusion statistics in controlled settings. However, these studies are confined to simple block or analytical geometries, and they also did not quantify a coronary-specific diameter-defined Strahler hierarchy or detailed order connectivity [37]. More recent work has explicitly targeted the coronary circulation by coupling synthetic tree growth to image-derived anatomy or global optimization principles. Jaquet et al. [18] extended base growth to multiple competing trees attached to segmented epicardial coronaries from CT images, filling the left ventricular myocardium with arteriolar scale vessels and reporting good agreement between synthetic and empirical morphometry in terms of diameter and length versus Strahler order. Mahmoudi et al. [19] combined coronary CT angiography, ferumoxytol-enhanced fractional myocardial blood volume maps, and an adaptive multistage CCO algorithm to generate subject-specific arterial microvascular networks with thousands of terminals per subject and showed strong correlations between synthetic morphometry and coronary cast-based models, along with plausible one-dimensional hemodynamics in swine and human data. Complementary non-CCO methods have used tissue demand-driven growth and global optimization in idealized or patient-specific cerebrovascular and coronary geometries to enforce supply demand balance and minimize composite costs related to pumping power and blood volume, achieving physiological pressure and radius distributions and energetically efficient trees [38–42]. In parallel, rule-based tree generators and image synthesis frameworks such as VascuSynth and stochastic L system models have focused on producing anatomically plausible networks and volumetric images for segmentation and registration validation rather than detailed hemodynamics or coronary-specific hierarchy [3, 43–45]. Beyond incremental growth formulations, anatomy-driven reconstruction studies have also generated large-scale coronary networks directly from detailed morphometric databases. Kaimovitz et al. [46] developed a stochastic geometric framework posed as a global optimization problem to reconstruct complete three-dimensional porcine coronary arterial trees down to the capillary level while preserving experimentally observed morphometry. This work was later extended to a full reconstruction of the entire porcine coronary vasculature including arterial, capillary, and venous networks, providing one of the most comprehensive anatomically consistent coronary network models for large-scale simulation studies [47, 48]. Together, these coronary-oriented frameworks demonstrate that vascular network generation can be constrained by image-based anatomy, perfusion imaging, tissue demand, energetic optimality, or morphometry constrained reconstruction, and can achieve realistic global morphometry and physiological pressure flow behavior in many cases.

Despite the progress made in generating realistic coronary arterial networks, several important gaps still remain. Specifically, in the hybrid model of [18], the intramural trees extend down to the arteriolar scale and, when mapped to the porcine diameter-defined Strahler hierarchy, generated segments span orders 4–9. Consequently, smaller arteriolar (orders < 4) and capillary levels are not represented, and tree growth is not robust for all epicardial outlets, with a subset of trees (62 out of 285) failing to reach their prescribed flow (often below ~ 80% of target), and a smaller subset failing to grow at all. In the multistage CCO framework of [19], synthetic networks with thousands of terminals per subject are compared with porcine morphometry by grouping segment diameters and lengths using conventional Strahler ordering, yet the resulting diameter- and length-order curves, while following the overall experimental trend, remain systematically offset from the empirical relations over several orders. Alternative demand-driven and globally optimized methods capture physiological pressure–flow relations and reduced energetic cost but represent microcirculatory supply through coarse spheres of influence or reduced-order segments rather than explicit arteriolar and precapillary hierarchies, and their coronary applications have not been validated against detailed coronary morphometry across all orders and connectivity levels. Across these CCO-based and non-CCO approaches there is therefore still no single framework that integrates CT-derived macrovascular anatomy and left ventricular geometry with physiologically consistent local bifurcation optimization and a mechanism that stabilizes the emergent diameter–order hierarchy under stochastic growth, while producing complete coronary arterial trees that can be analyzed in terms of both diameter-defined Strahler order and order connectivity. These differences between the present framework and the two most closely related image-based methods are summarized in Table 3.

To address these limitations, the present framework extends the conventional CCO methods in three main directions. ***First***, both the admissible growth domain and the proximal coronary scaffold are derived directly from subject-specific CT images [21]. Specifically, a segmentation-based LV mask and associated Euclidean distance field constrain candidate segments to the myocardial wall with prescribed clearance from the endocardial and epicardial surfaces, while a segmentation-derived epicardial coronary tree provides the initial topology and diameters so that growth proceeds as an anatomically grounded continuation of the imaged macro-vasculature into sub-resolution territories. ***Second***, local bifurcations are generated using a Kamiya-type optimization seeded with HK-type daughter radii and a flow-weighted bifurcation point, yielding branch locations and diameter ratios that are closer to a physiological flow–radius balance than those obtained from symmetric or purely geometric initial guesses. ***Third***, the growth of the coronary tree is governed by a hierarchy-aware, stage-wise branching schedule that progressively shifts branching toward more distal segments as the tree fills the domain, thereby stabilizing the emerging diameter-order hierarchy under stochastic growth. Together, these new features enable the generation of coronary arterial trees in which subject-specific macrovascular anatomy and synthetic microvascular extensions jointly exhibit realistic branching patterns, order–order connectivity, and diameter-order behavior. Runtime was also comparable to the conventional CCO implementation on the same domains, indicating that the hierarchy-aware constraints and flow-based bifurcation initialization do not materially increase cost; the practical advantage of the framework over conventional CCO therefore lies in the fidelity and reproducibility of the generated networks rather than in runtime. Relative to prior image-based coronary generators, which report multi-day generation times ([18] as a Python prototype, [19] approximately two days per subject), the present compiled implementation is more efficient (Table 3).

### Hierarchical Organization, Connectivity, and Diameter Relationships

Beyond visual inspection of single trees, we assessed whether the HCT-CCO framework produces a stable, physiologically meaningful order-level hierarchy under stochastic growth. On a fixed LV domain, it reproduced the banded, near-diagonal order–order connectivity of the experimental matrix [13] with substantially higher precision, recall, and F1 score than the conventional formulation, and consistently produced a monotonic, reproducible diameter–order hierarchy spanning DDS orders 1–11 with markedly reduced inter-run variability. In contrast, the conventional formulation yielded irregular, poorly reproducible hierarchies that were more sensitive to stochastic branching history. These findings indicate that the hierarchy-aware branching rules promote a coherent order-level organization that is more consistent with experimental coronary morphometry than conventional CCO.

Although in this study we calibrated the hierarchy model to the porcine diameter-defined Strahler hierarchy and connectivity matrix of [13], the same strategy is, in principle, species-agnostic. The connectivity matrix, diameter–order relations, length statistics, and physiological targets for pressures and flows can be replaced by morphometric and functional data from other species, while retaining the same optimization and branching machinery. This would allow the framework to be configured for different hearts by re-parameterizing the hierarchy model to the corresponding connectivity and diameter–order relationships and generating synthetic coronary trees consistent with the size, branching pattern, and perfusion demands of each species.

### Subject-Specific Networks, Transmural Distribution, and Implications

Applying the HCT-CCO algorithm to five CT-derived porcine LV geometries and one synthetic case [21], we show it can be used to generate large scale, subject-specific coronary networks that are in quantitative agreement with experimental measurements of coronary morphometry. In these networks, vessel diameters follow a consistent diameter–order relationship, and the arterial hierarchy extends from DDS order 1 to 11, indicating that the framework reliably reproduces the expected depth of the coronary tree across different LV shapes. The transmural analysis of leaf vessel distribution shows a near balanced but slightly epicardial dominant pattern across the LV wall. On average, 47.87% of leaf vessels were located in the inner 50% volume of the wall and 52.13% in the outer 50%, with five of the six cases showing higher vessel counts in the outer half. This modest epicardial predominance should be interpreted with caution. New terminals are sampled throughout the myocardial wall, but the base tree and its larger proximal vessels lie on the epicardial surface, so the initialization is spatially biased toward the epicardium and may influence the resulting terminal distribution; the observed bias therefore cannot be attributed to physiology alone. The pattern is qualitatively consistent with morphometric reports of higher epicardial microvascular density [49, 50]. However, those studies quantified capillary density, whereas the terminal vessels generated here are precapillary arterial branches rather than capillaries, so the comparison is only indirect. We therefore do not present the transmural distribution as an independent physiological validation and separating the methodological and physiological contributions would require a dedicated analysis with subject-specific perfusion data. Beyond morphometry, the generated networks are directly usable for biomechanical analysis: the hemodynamic assessment in Sect. “Hemodynamic plausibility” solves a representative network for segment flows, pressures, and wall shear stress, demonstrating that the trees provide a ready substrate for coronary hemodynamic and perfusion modeling rather than a purely geometric reconstruction.

### Limitations and Future Directions

Although the framework reproduces key morphometric and transmural trends and is tightly integrated with image-derived anatomy, several limitations remain. ***First***, the imaging pipeline relies on manual coronary segmentation, introducing operator variability and limiting scalability to larger cohorts. ***Second***, terminal flow is prescribed uniformly, with each terminal segment receiving equal flow. This is a deliberate modeling choice: equal terminal flow has been shown to reproduce physiological ranges of perfusion heterogeneity, including fractal dimension and nearest-neighbor correlation, whereas spatially weighted flow prescriptions shift these patterns toward hypoperfusion [15, 16]. It does not, however, capture subject-specific or regional perfusion heterogeneity, and subject-specific physiological flow boundary conditions are not yet imposed, so the hemodynamic state is calibrated to canonical scaling behavior rather than individualized flow distributions. A natural extension is to replace the uniform prescription with non-uniform terminal flows derived from perfusion imaging, as demonstrated for coronary networks using fractional myocardial blood volume maps [19]. In its present anatomy-driven form with a uniform terminal flow prescription, the framework represents the healthy microvascular organization. Because the terminal flow prescription is a supplied input, it can represent acute changes in microvascular function, such as microvascular obstruction, in an otherwise healthy network. It cannot, however, reproduce disease-related changes in microvascular morphology, which would require histological data. Its key role is therefore to provide an anatomically realistic coronary network on which such disease scenarios can be simulated. ***Third***, the validation is based on porcine morphometry; although porcine hearts provide a well-established analog, species-specific differences in animal coronary scaling and transmural perfusion may still exist. Validating the framework for humans will require reference data, such as ex vivo human coronary casts, micro-CT imaging, and histological morphometry to test the vessel hierarchy, together with clinically measured coronary flow and regional myocardial perfusion to test the functional behavior. ***Finally***, this study was deliberately scoped to the left coronary system within the left ventricular myocardium: the base trees comprise the LAD and LCx, which supply the majority of the LV free wall. The right coronary artery was not used as a base tree, a deliberate scope choice rather than an imaging or segmentation limitation. The RCA primarily perfuses the right ventricle and, in a right-dominant heart, the inferior and posterior LV wall; that LV territory is therefore populated here by extensions of the left coronary system rather than by RCA-derived vessels, and coronary dominance is not explicitly represented. Because the growth domain and base tree are supplied inputs, the framework is not specific to the left system or to the LV and extends naturally to the RCA and right ventricle: a segmented RCA base tree and its perfusion territory, together with the RCA connectivity matrix and diameter-order relations reported separately in the same porcine benchmark [13], would allow RCA-derived territories and left- or right-dominant patterns to be represented within the same machinery. Extension to combined left and right ventricular whole-heart networks, incorporating these RCA data, is the subject of ongoing follow-up work.

In future work, we are also actively developing a deep-learning pipeline that automates the full preprocessing chain, taking a computed tomography angiography (CTA) volume as its only input and producing both the LV myocardial domain and the coronary base tree without manual intervention. This automation is intended to remove operator variability and enable large scale, operator-independent generation of base trees across larger cohorts. Because the growth machinery is independent of the specific morphometric targets, the framework can be applied to human anatomy by replacing the porcine connectivity matrix and diameter-order relations of [13] with corresponding human coronary morphometric data and by re-parameterizing the transmural perfusion pattern accordingly; the automated pipeline currently under development operates directly on human CTA data, providing a natural route toward human-specific coronary network generation. Beyond the morphometric validation and the preliminary hemodynamic check reported here, an important next step is functional validation of the generated networks against measured or imaging-derived hemodynamics, comparing predicted pressure-flow relationships, regional myocardial blood flow, microvascular resistance, and perfusion distributions with experimental measurements or perfusion imaging. The same anatomy underpins clinically oriented applications, including computation of fractional flow reserve (FFR) and other pressure-flow indices for diagnostic assessment and, in combination with perfusion imaging, prognostic evaluation of coronary disease and its microvascular component. Coupling the algorithm with hemodynamic solvers would allow joint optimization of vascular geometry and the resulting pressure–flow fields, rather than relying solely on geometric criteria. Integration of adaptive radius laws or wall-stress–dependent feedback would extend realism toward mechanobiological growth modeling, in which vessels’ diameter and branching patterns co-evolve with their mechanical and perfusion environment.

### Conclusions

In summary, this work introduces an HCT-CCO algorithm, which integrates subject-specific LV anatomy, segmentation-derived epicardial base trees, hierarchy-aware growth, and a DDS order-based analysis pipeline to generate coronary arterial networks with reproducible hierarchical organization and experimentally consistent diameter-order connectivity. The framework produces trees whose order-wise diameter statistics align with established morphometric benchmarks, and the resulting diameter-order relationships match the expected hierarchy across DDS order 1–11. Application to both synthetic and subject-specific LV geometries demonstrates that the framework can produce anatomically confined, morphometrically controlled coronary trees suitable for downstream physiological modeling. These developments advance CCO-based coronary modeling from idealized domains toward quantitatively validated, subject-specific networks, providing a foundation for future studies of coronary hemodynamics and perfusion in subject-specific geometries.

## Figures and Tables

**Fig. 1:**
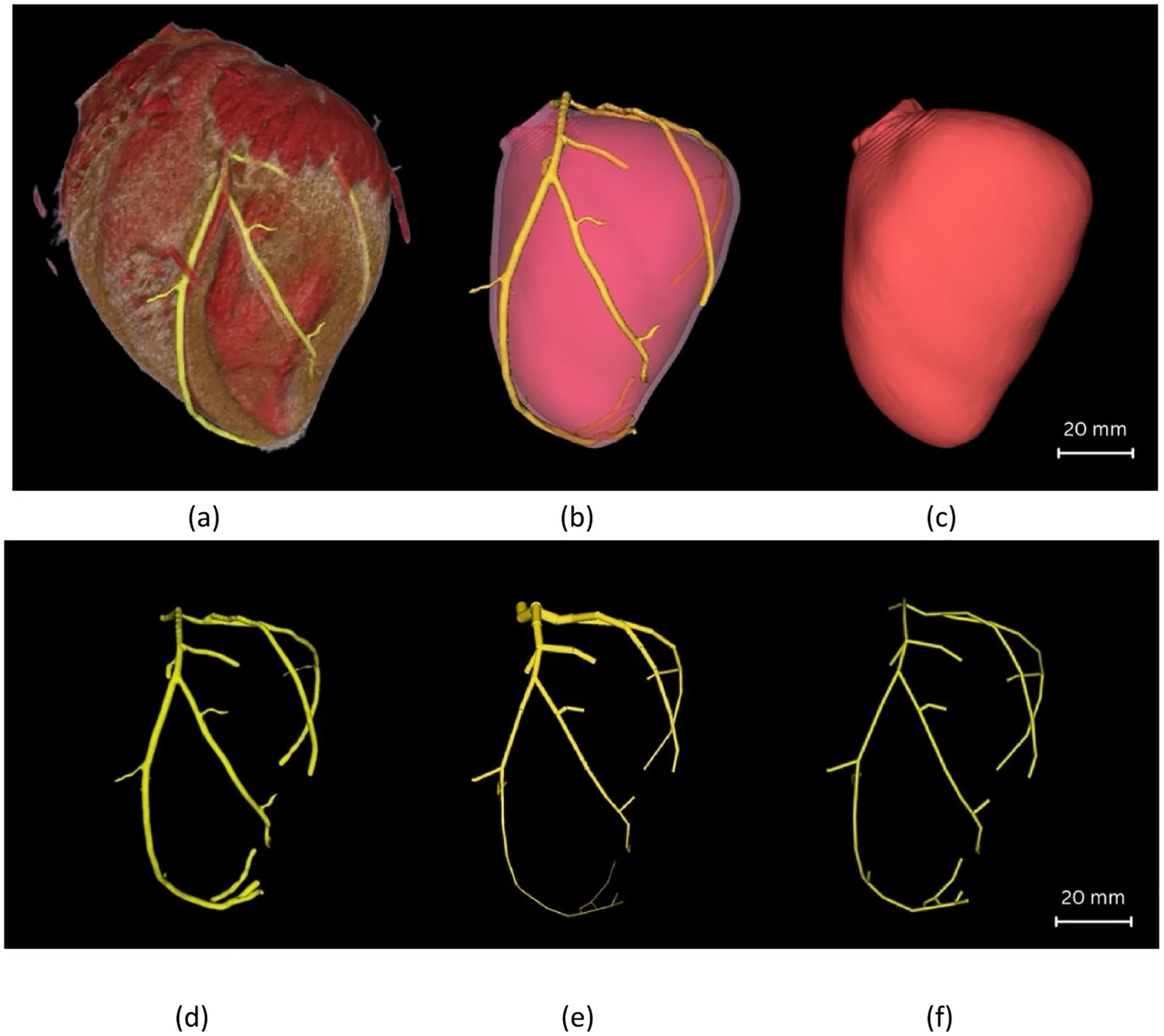
HCT-CCO preprocessing with CT-based segmentation. **a** Volume-rendered CT of the heart showing visible epicardial coronaries, **b** manually segmented LV myocardial wall with epicardial coronary arteries overlaid, **c** Cleaned LV myocardial wall, **d** Cleaned epicardial coronary volume segmentation, **e** Simplified vessel geometry with adjusted lumen radii used for centerline extraction and **f** Coronary centerline graph with preserved bifurcations, used as the base tree for HCT-CCO growth

**Fig. 2:**
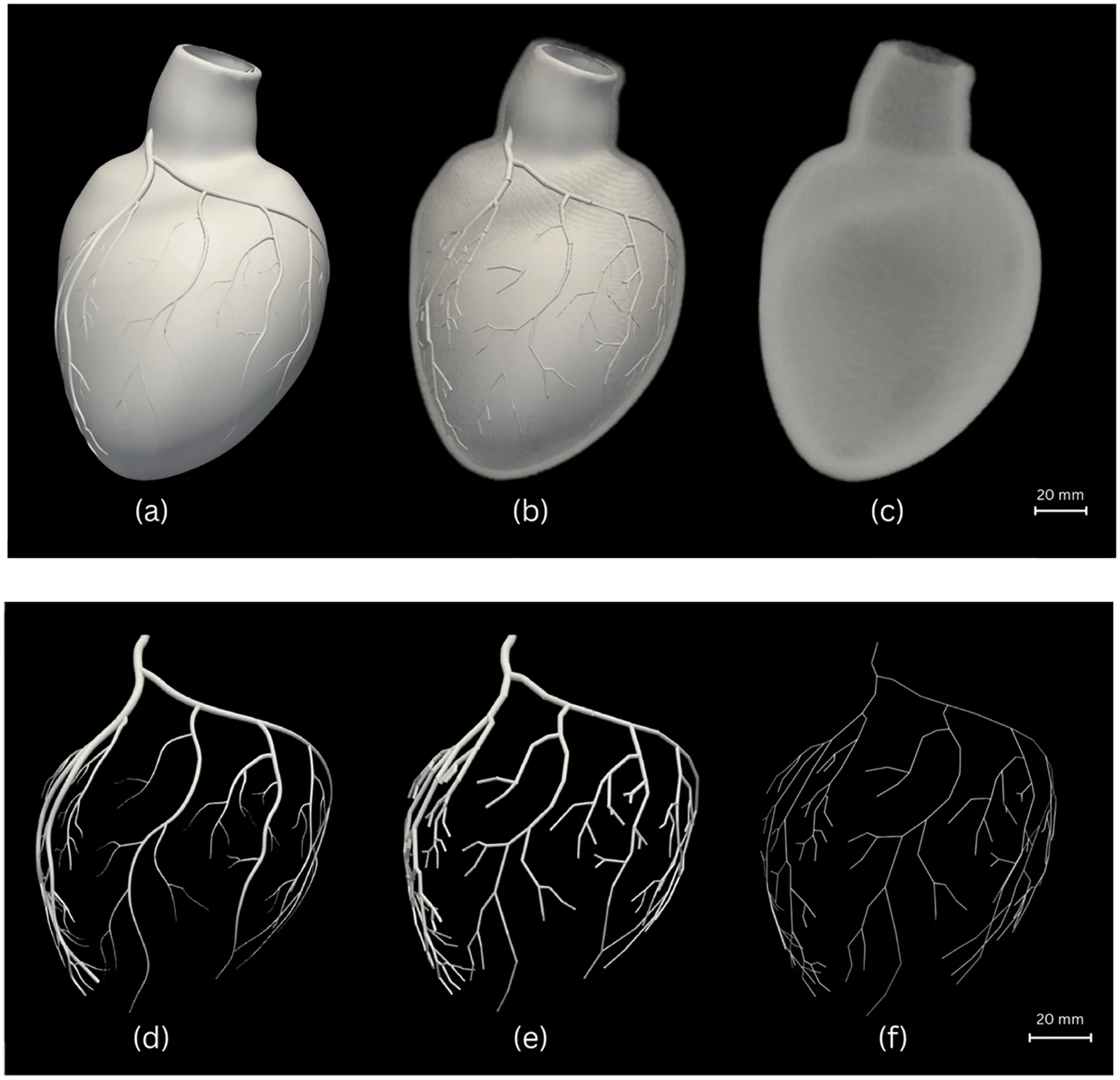
CT-derived LV domain and coronary base tree network preprocessing workflow. **a** Segmented LV myocardial wall with epicardial coronary arteries overlaid, **b** Rasterized LV myocardial wall volume obtained from epicardial and endocardial surfaces, **c** Final LV myocardial growth domain Ω, **d** Cleaned epicardial coronary volume segmentation, **e** Simplified vessel geometry with adjusted lumen radii used for centerline extraction, and **f** Coronary centerline graph with preserved bifurcations, used as the base tree for HCT-CCO growth

**Fig. 3:**
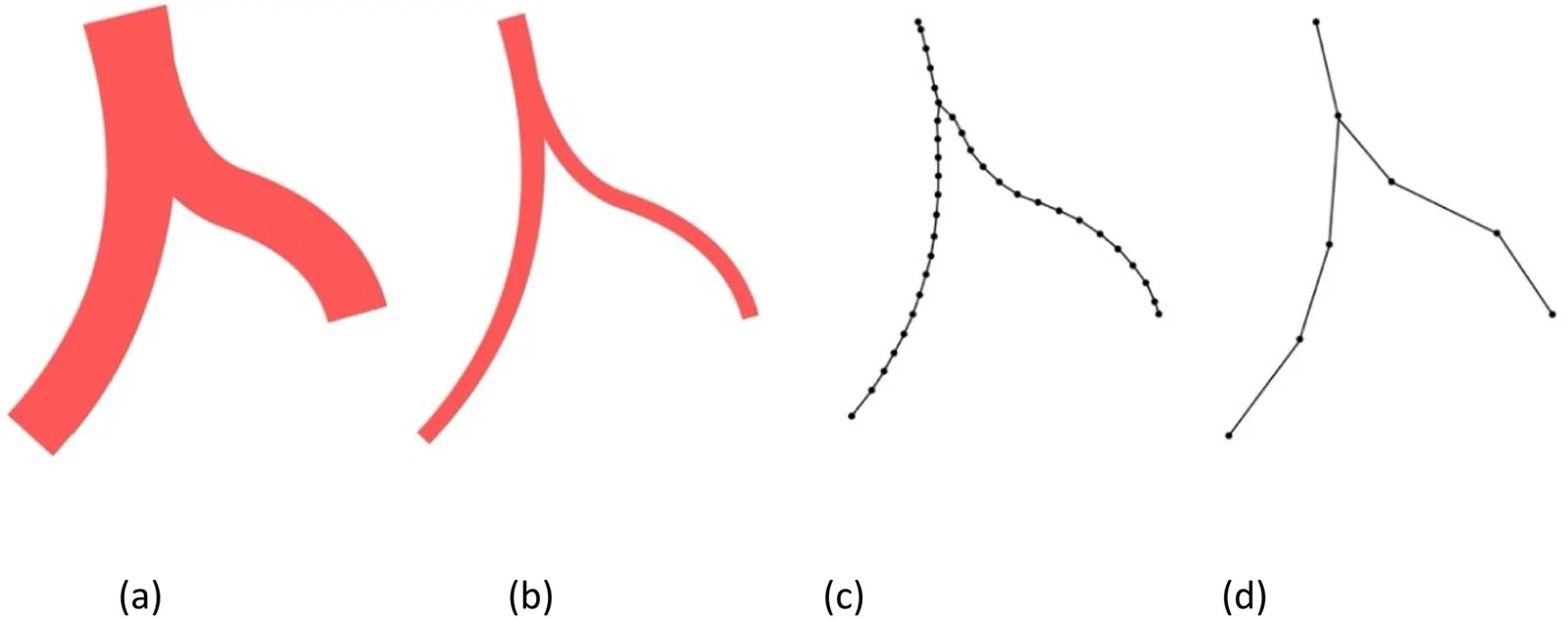
Vessel preprocessing and centerline graph construction. **a** Segmented vessel volume, **b** Thinned vessel used for stable centerline extraction, **c** Extracted vessel centerlines, and **d** Simplified centerline graph with preserved bifurcations

**Fig. 4:**
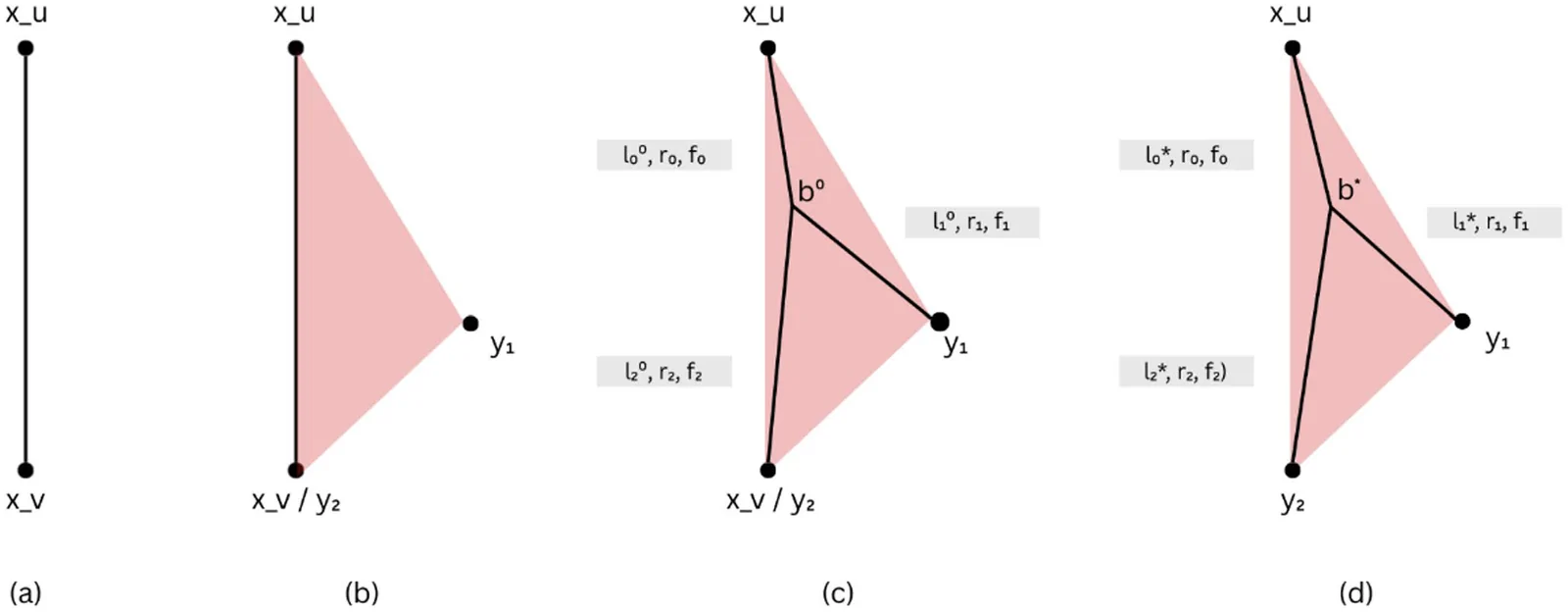
Local geometric optimization of a bifurcation. **a** Existing host segment with upstream and downstream nodes $x_{u}$ and $x_{v}$ ($x_{v}$ *is* later denoted ${\text{y}}_{2}$ for consistency with daughter notation); **b** introduction of a new terminal candidate $y_{1}$; **c** analytic initialization of the bifurcation at $b^{\left(0\right)}$, defining the parent and daughter segments with lengths ${l_{0}}^{\left(0\right)},{l_{1}}^{\left(0\right)}$ *and* ${l_{2}}^{\left(0\right)}$ corresponding radii and flows $\left(r_{i},f_{i}\right)$; and **d** the optimized bifurcation configuration a*t* $b^{*}$, yielding the final segment lengths ${l_{0}}^{*},{l_{1}}^{*}$, *and* ${l_{2}}^{*}$

**Fig. 5:**
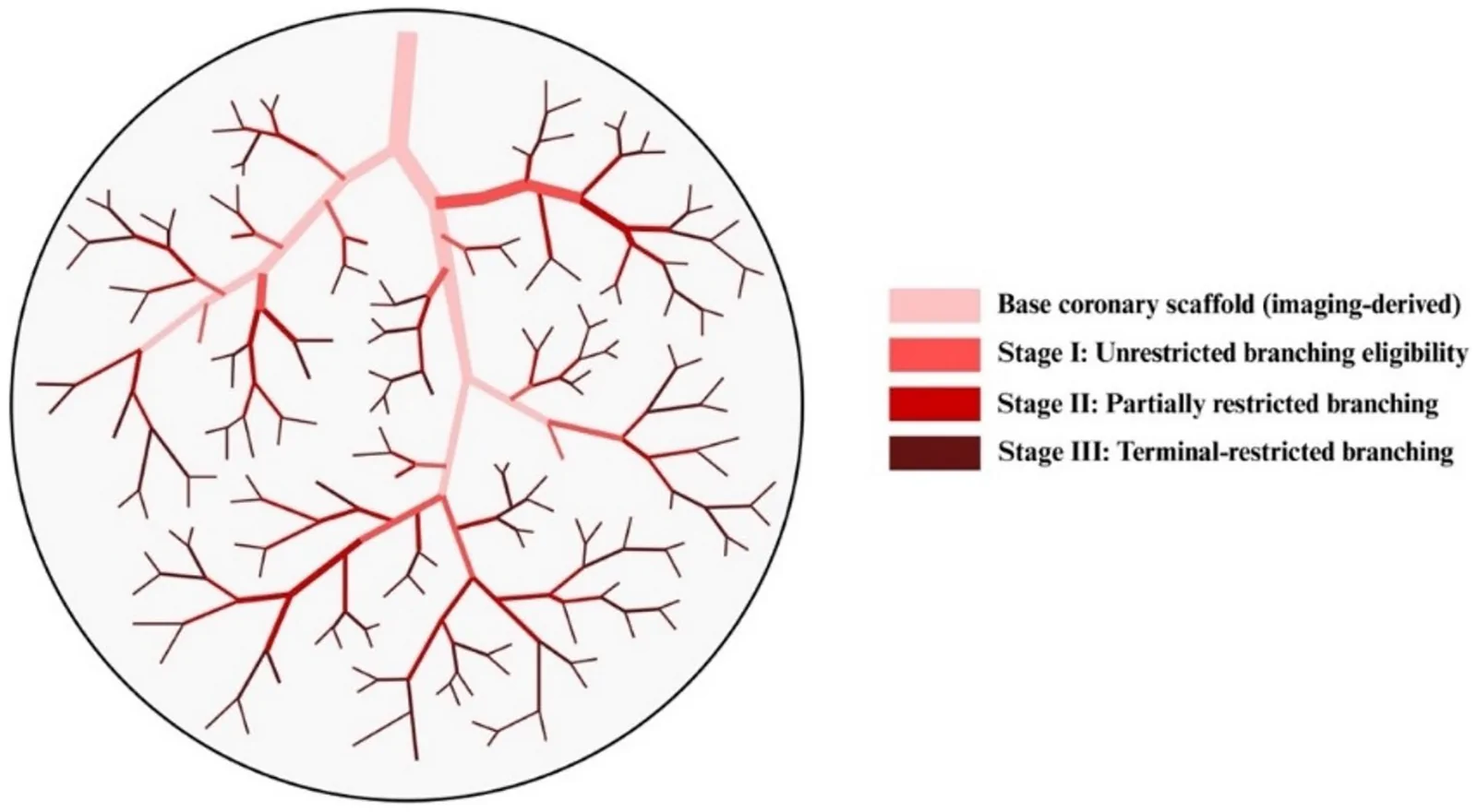
Illustration of the hierarchy-aware, stage-wise branching strategy

**Fig. 6:**
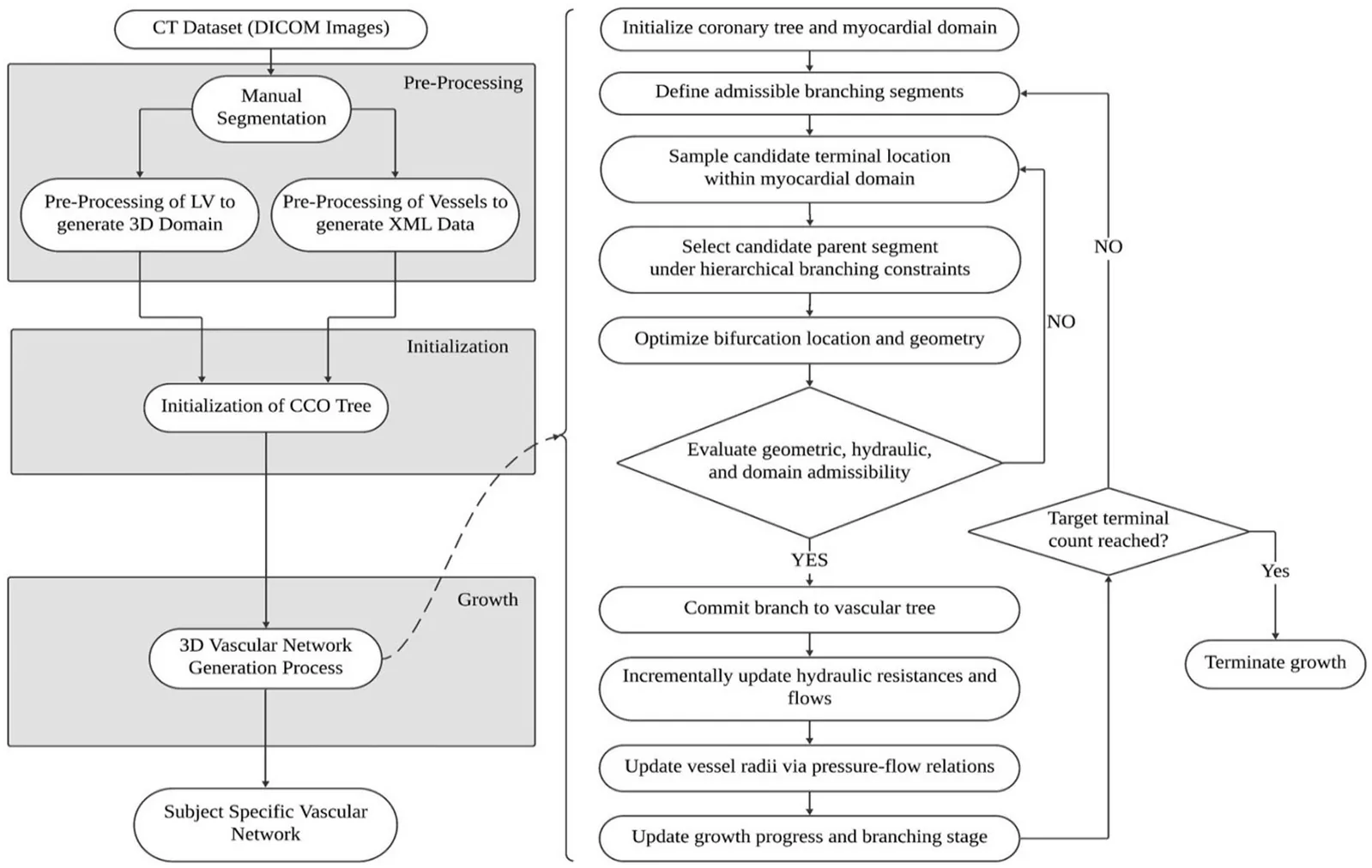
Block diagram of preprocessing, initialization, and constrained constructive optimization workflow

**Fig. 7:**
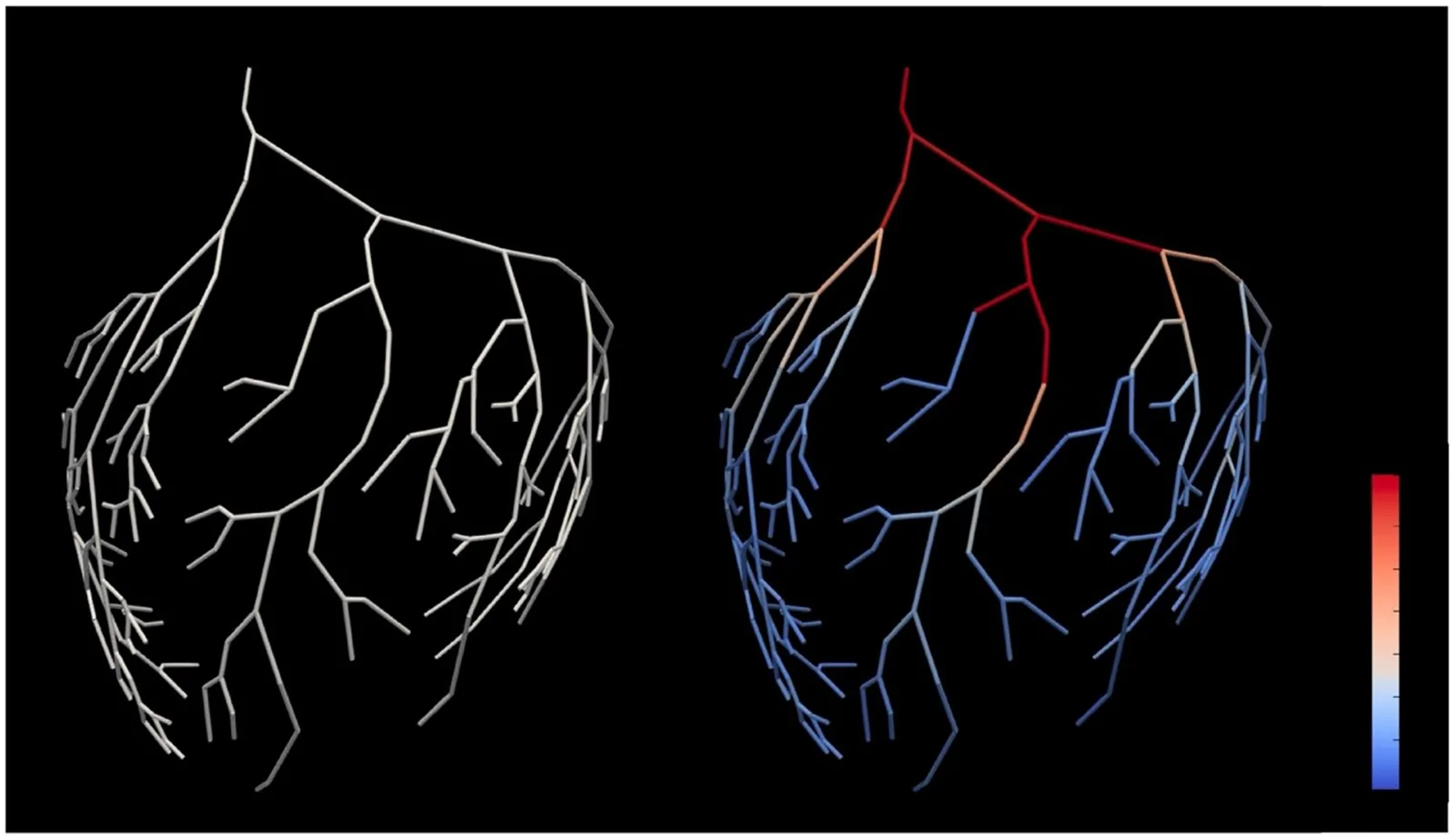
Centerline graph with radius values (left) produced by the line-graph generation pipeline; corresponding vessel orders (right) after implementing diameter-defined Strahler ordering

**Fig. 8:**
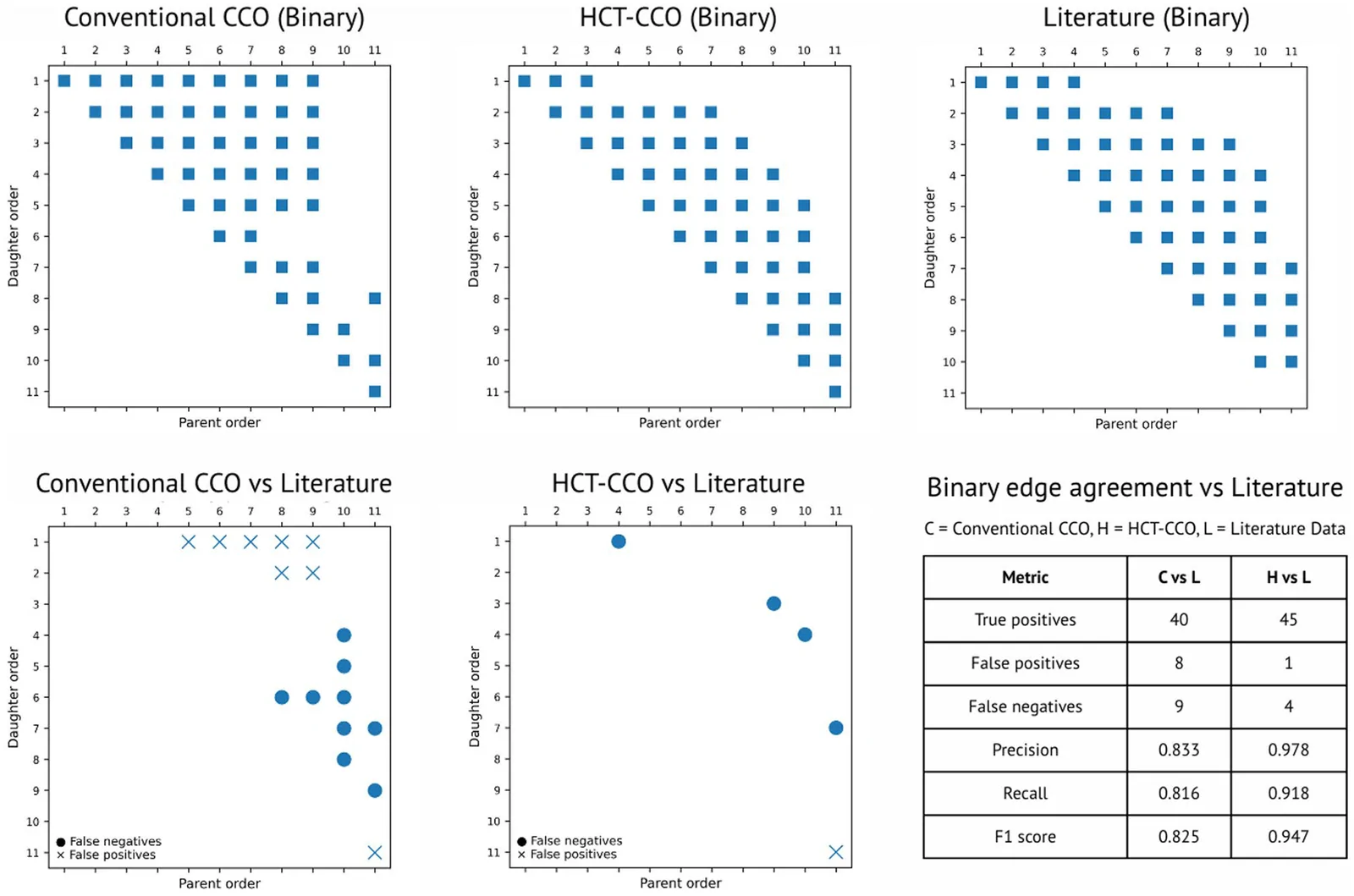
Binary order–order connectivity pattern for conventional CCO and HCT-CCO algorithm compared with experimental measurements (directed adjacency parent to daughter)

**Fig. 9:**
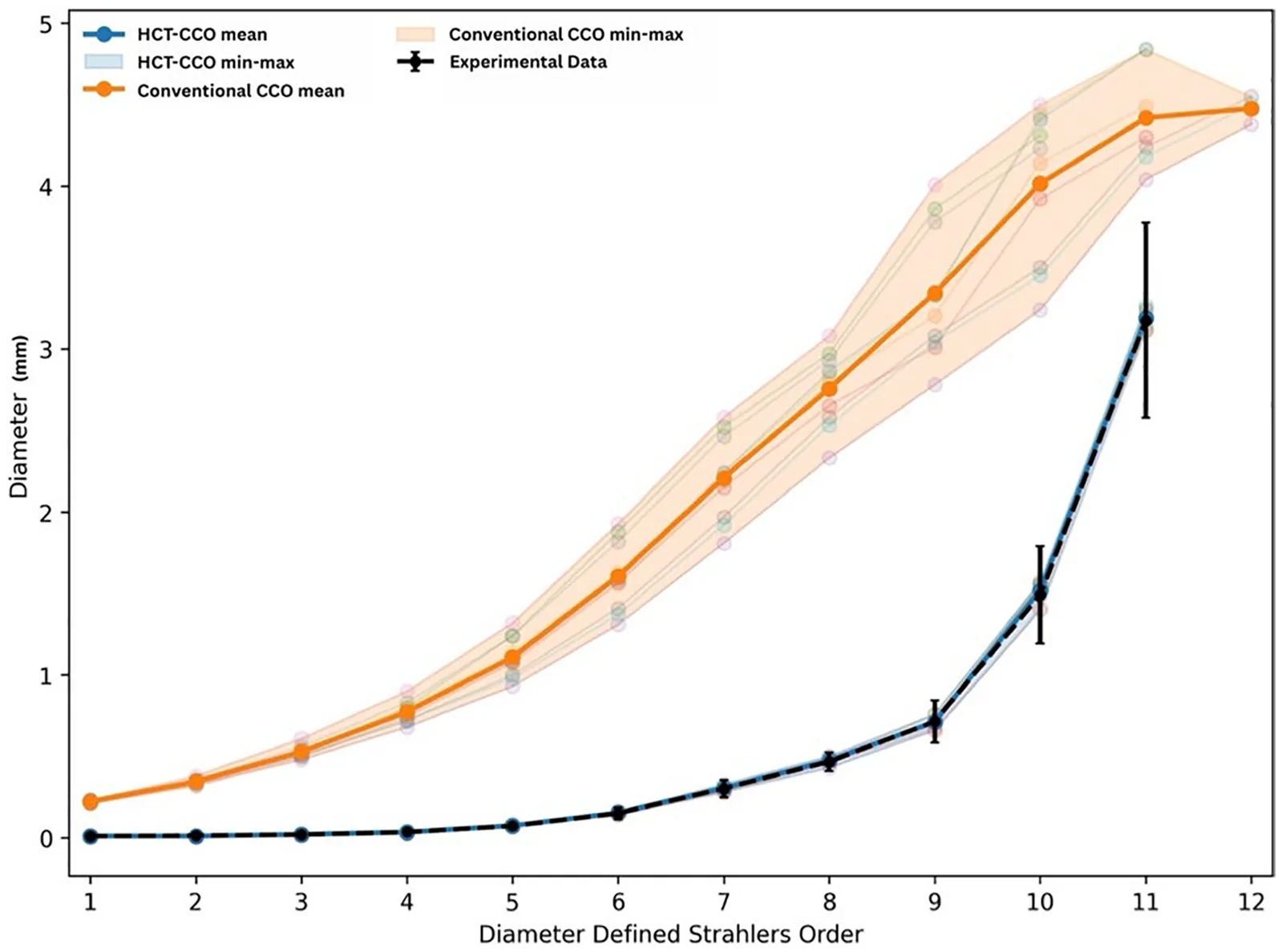
Comparative diameter–order relationships for conventional CCO and HCT-CCO across ten independent realizations on the same LV domain. The vertical axis shows mean vessel diameter (mm) on a linear scale, and the horizontal axis the diameter-defined Strahler (DDS) order. Both conventional CCO and HCT-CCO trees were ordered using the same DDS procedure, with the root anchored to the top order. Solid lines denote the mean across the ten realizations, shaded bands the minimum-to-maximum range across realizations, and the black markers the experimental porcine data [13]

**Fig. 10:**
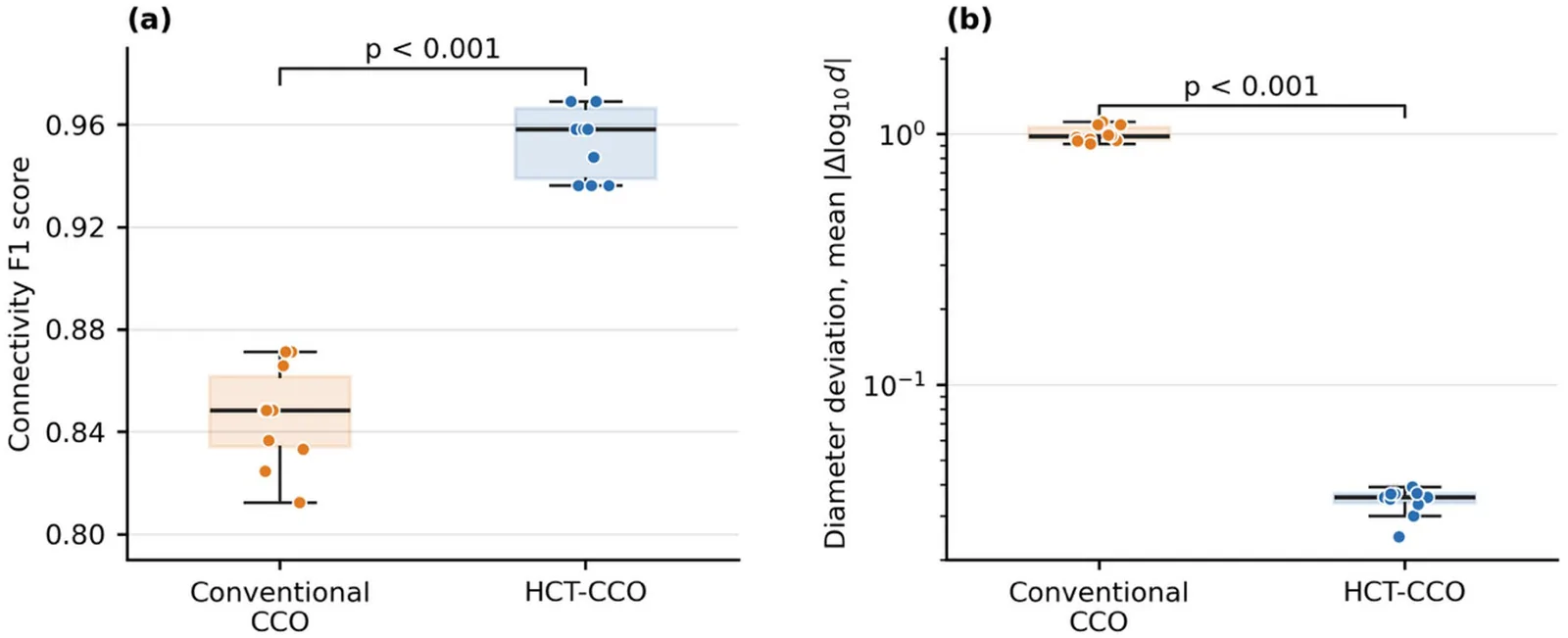
Statistical comparison of conventional CCO and HCT-CCO across ten independent realizations on the same LV domain. **a** Connectivity F1 score against the binarized literature matrix [13]. **b** Diameter-order deviation from the porcine benchmark, computed as the mean absolute log_10_ difference across orders 1–11 (log scale). Boxes show median and interquartile range; points are individual realizations. Both distributions are completely separated (two-sided Mann–Whitney U test, p<0.001; Cliff’s delta = 1.0)

**Fig. 11:**
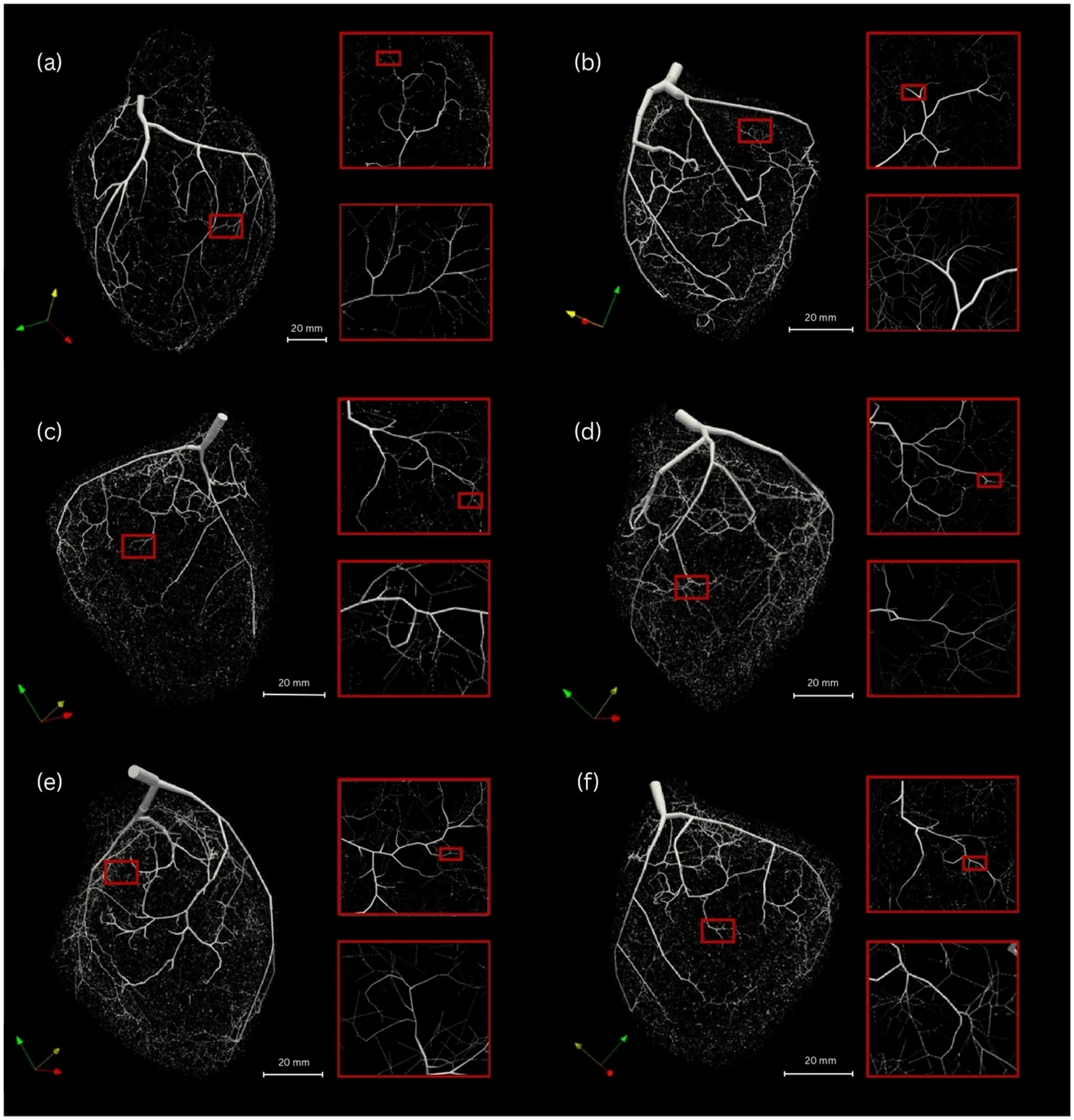
Vascular networks generated using the HCT-CCO algorithm for all cases (**a–f**). For each case, an inset shows a magnified view of a selected local region, highlighting the same vessels at higher spatial resolution

**Fig. 12:**
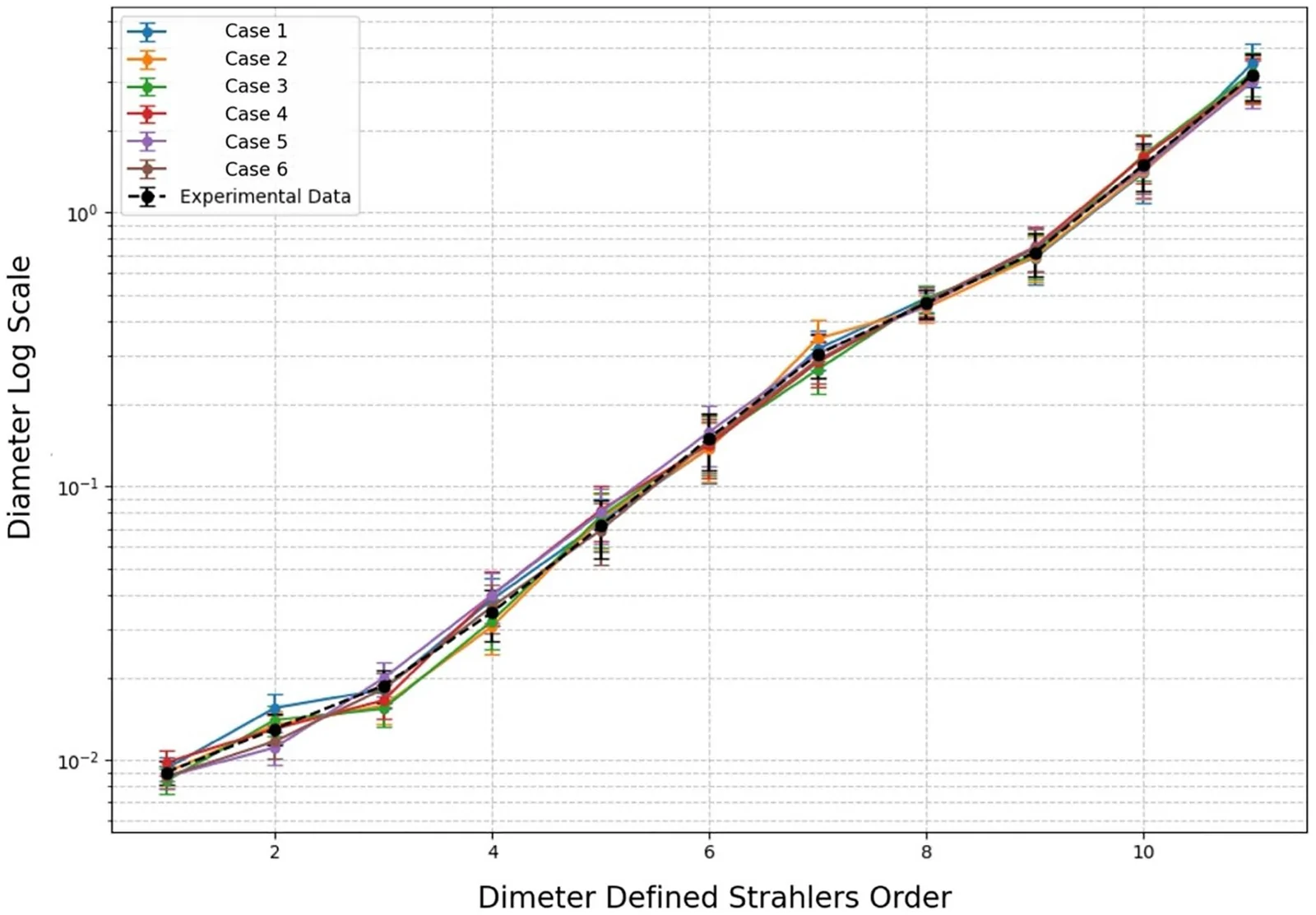
Semi-log plot (Y-axis) of diameter versus Strahler order (X-axis) with standard deviation bands for all six cases; dashed line indicates mean value from the experiment data

**Fig. 13:**
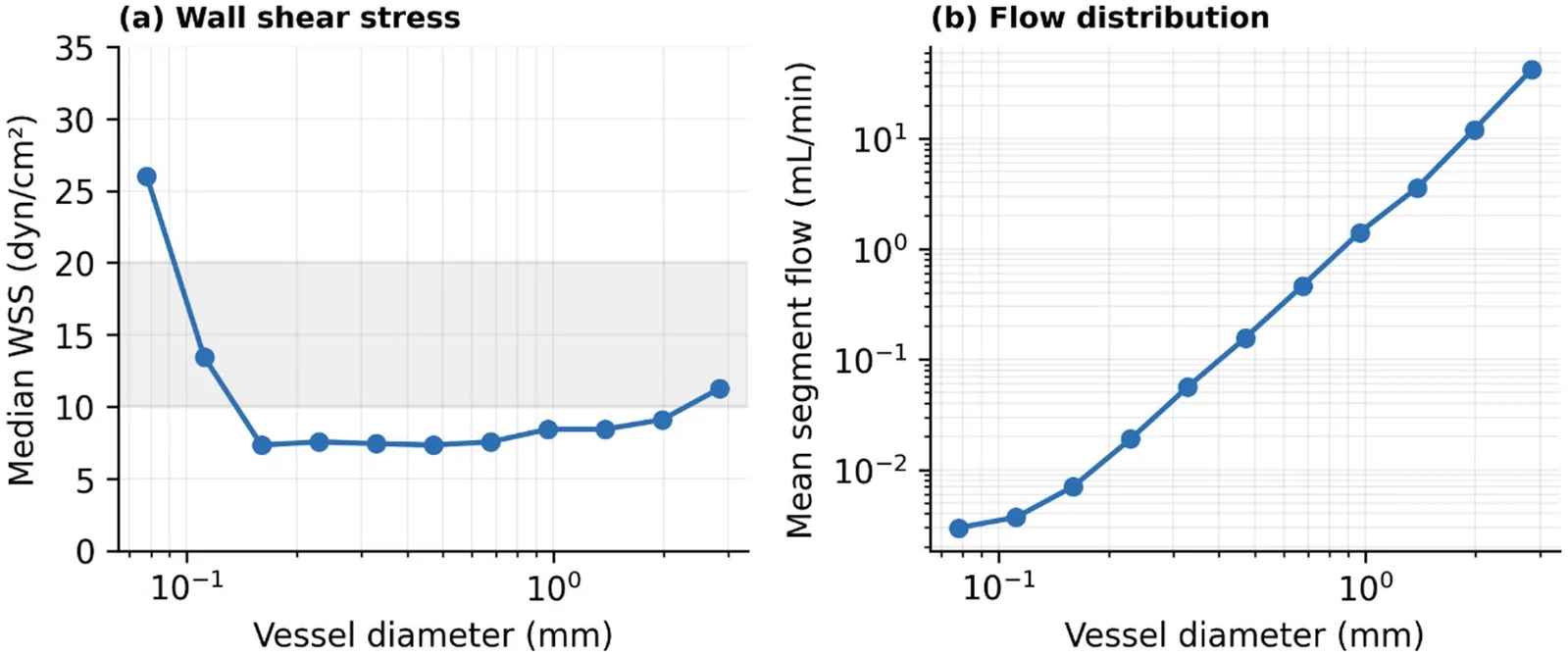
Hemodynamic check on a representative generated network. **a** Median wall shear stress versus vessel diameter, remaining within the physiological range (shaded band, 10 to 20 dyn/cm^2^). **b** Mean segment flow versus vessel diameter. Total flow was calibrated to a physiological perfusion density of 3.5 mL/min/g under a 100 mmHg inlet perfusion pressure

**Table 1:** Principal model parameters, their values, rationale or source, and expected sensitivity of the results

Parameter	Value	Rationale/source	Expected sensitivity
HK bifurcation exponent, γ	2.33	HK coronary scaling law [20]	Moderate; sets daughter-to-parent radius ratios
Inlet radius cap	1.7 mm	Proximal LAD/LCx caliber [25]; largest DDS order [13]	Low; caps the root only, rarely binds
Taper-floor factor	0.7	Just below symmetric daughter/parent ratio (HK ≈ 0.74 [20], Murray ≈ 0.79 [29], Finet ≈ 0.68 [36])	Low; lower bound, rarely binds
Minimum boundary clearance	5 voxels (~5 mm)	Confines vessels within the myocardial wall	Low to moderate; controls wall confinement
Terminal spacing	Adaptive, 4π⋅r_perf3/3k_term)^(1/3)	Characteristic perfusion spacing; decreases as the tree fills	Derived, not a free parameter; sets terminal density
Maximum segment length	25 mm	Prevents non-physiologic long vessels	Low; upper bound, rarely binds
Minimum segment radius	0.005 mm	Prevents sub-resolution vessels [13]	Low
Branching schedule thresholds	f=0.10, 0.35	Vessel-count distribution of the coronary tree [13] (Sect. “Hierarchy-Aware Branching Strategy”)	Moderate; governs order-order connectivity
Perfusion (inlet) pressure	100 mmHg	Standard CCO boundary condition [15, 37]	Sets pressure scale; low effect on morphometry
Perfusion pressure drop	~ 37 mmHg (terminal ~ 63 mmHg)	Standard CCO boundary condition (terminal ~ 60 mmHg) [15, 37]	Sets gradient; low effect on morphometry
Blood viscosity	3.6 cP	Standard CCO blood viscosity [37]	Scales resistances and radii uniformly
Number of terminals	Case-specific (~1.2–2.0 × 10^4^)	Target perfusion/tree density	Moderate to high; sets density and maximum resolved order

**Table 2.** Transmural distribution of order 1 vessels across LV wall layers (inner/outer 50%) for each case

Case	Total number of leaf vessels	Inner 50% segment (endocardial layer)	Outer 50% segment (epicardial layer)
1	17,935	45.83% (8,220)	54.17% (9,715)
2	13,926	52.26% (7,278)	47.74% (6,648)
3	12,241	47.52% (5,817)	52.48% (6,424)
4	18,619	46.99% (8,749)	53.01% (9,870)
5	19,690	47.95% (9,442)	52.05% (10,248)
6	16,468	46.65% (7,682)	53.35% (8,786)

**Table 3:** Comparison of the HCT-CCO framework with the two most closely related recent image-based coronary tree generation methods

Feature	Jaquet et al. [18]	Mahmoudi et al. [19]	HCT-CCO (this work)
Algorithm	Hybrid image-based synthetic forest (multi-tree CCO)	Multistage adaptive CCO	Hierarchy-aware CT-driven CCO
Imaging/base data	Cardiac CT, segmented epicardial coronaries	FE-MRI (coronary MRA + fMBV maps)	Cardiac CT, segmented LV wall + epicardial coronaries
Subjects	6 patients	3 swine, 4 volunteers, 1 IHD patient	1 synthetic + 5 porcine
Terminal (leaf) vessels per model	6000	6000	~ 1.2 to 2.0 × 10^4^ (Table 2)
Smallest resolved diameter	~170μm	~200μm	~10μm
DDS Strahler order range	4 to 9 (segments)	4 to 11	1 to 11
Diameter–order agreement	R^2^ > 0.99 (diameter)	Spearman r = 0.99 (diameter)	R^2^ = 0.998 (vs Kassab [13])
Connectivity matrix validated	No (imposed as input)	Not reported	Yes, F1 = 0.95 ± 0.01
Reproducibility across runs	flow r = 0.92 across seeds	terminal-diameter CoV = 0.008	F1 = 0.95 ± 0.01; Mann–Whitney, Cliff’s δ=1.0
Growth robustness	62/285 sub-trees (22%) below 80% target flow	Not reported	6/6 cases reached orders 1 to 11, no failures
Implementation / runtime	Python prototype, several days per model	~ 2 days per subject, single CPU core	compiled C+ +, ~ 15 to 20 h per case

Values are as reported in the respective studies, except where computed from the present data. *FE-MRI* ferumoxytol-enhanced MRI, *fMBV* fractional myocardial blood volume, *DDS* diameter-defined Strahler, *CoV* coefficient of variation

## Data Availability

The HCT-CCO source code, including a worked example (myocardial domain and epicardial base tree) and the postprocessing pipeline, is publicly available at https://github.com/Sujal-vajire/HCT-CCO. The CT-derived geometries and processed vascular network data supporting the findings of this study are available from the corresponding author upon reasonable request.
